# Stimulus-responsive smart bioactive glass composites for repair of complex tissue defects

**DOI:** 10.7150/thno.104944

**Published:** 2025-01-02

**Authors:** Yulian Yang, Yonghao Qiu, Cai Lin, Xiaofeng Chen, Fujian Zhao

**Affiliations:** 1 Stomatological Hospital, School of Stomatology, Southern Medical University, Guangzhou 510280, PR China.; 2 Department of Burn, the First Affiliated Hospital of Wenzhou Medical University, Wenzhou 325000, PR China.; 3 Department of Biomaterials, School of Materials Science and Engineering, South China University of Technology, Guangzhou, Guangdong 510641, PR China.; 4 National Engineering Research Center for Tissue Restoration and Reconstruction, Guangzhou, Guangdong 510006, PR China.

**Keywords:** Smart, Bioactive glass, Stimulus-response, Complex tissue defect repair, Microenvironment

## Abstract

Smart biomaterials with active environmental responsiveness have attracted widespread attention in recent years. Previous studies on bioactive glass (BG) have mainly focused on the property of bioactivity, while little attention has been paid to the property of smart response of BG. Herein, we propose the concept of Smart Bioactive Glass Composites (SBGC) which are capable of actively responding to the endogenous disease microenvironment or exogenous physical stimuli, thereby enabling active treatment of tissue defect sites and ultimately promoting tissue regeneration. In this review, the response characteristics of SBGC to different internal and external environments are described. Subsequently, the applications of SBGC in complex tissue defect repair of tumors, infections, and diabetes are reviewed. By deeply analyzing the recent progress of SBGC in different fields, this review will point out the direction for the research of next-generation BG.

## Introduction

Bioactive glass (BG) is a typical bioactive material that promotes tissue regeneration and repair by activating gene expression through the dissolution of bioactive ions. Due to its excellent biological activity, BG has been widely used in the repair of tissue defects, such as bone, skin and teeth. However, when tissue defects are combined with other diseases (*e.g.*, refractory infections, tumors), the repair effect of BG is not ideal as it can only provide a basic therapeutic effect.

In recent years, smart repair materials that actively respond to the environment have become a hot research topic. Smart bioactive glass composites (SBGC) with smart properties have also been reported in various fields. It has been attempted for the treatment of complex diseases such as tissue defects combined with tumors, infectious diseases, acute bleeding, diabetes, and its complications. SBGC is mainly characterized by its ability to actively respond to the endogenous disease microenvironment (*e.g.*, pH, reactive oxygen species, specific enzymes, *etc*.) and exogenous physical stimuli (*e.g.*, magnetic field, light, electrical stimulation,* etc.*), thus enabling active treatment of the lesion and promoting tissue regeneration. This targeted responsiveness allows the SBGC to meet different treatment needs effectively, providing more precise control and customized treatment options. In addition, compared with other smart biomaterials, SBGC has specific advantages including bioactive ion dissolution, gene activation, and mineralization properties. Some summaries of BG research progress have been reported with the deepening of research. However, previous reviews on BG have mostly focused on its preparation method^1^, therapeutic effects^2^,^3^, molecular mechanisms^4^, and translational applications^5^. Studies related to the use of SBGC for complex tissue repair have not yet been summarized.

**Figure 1:**
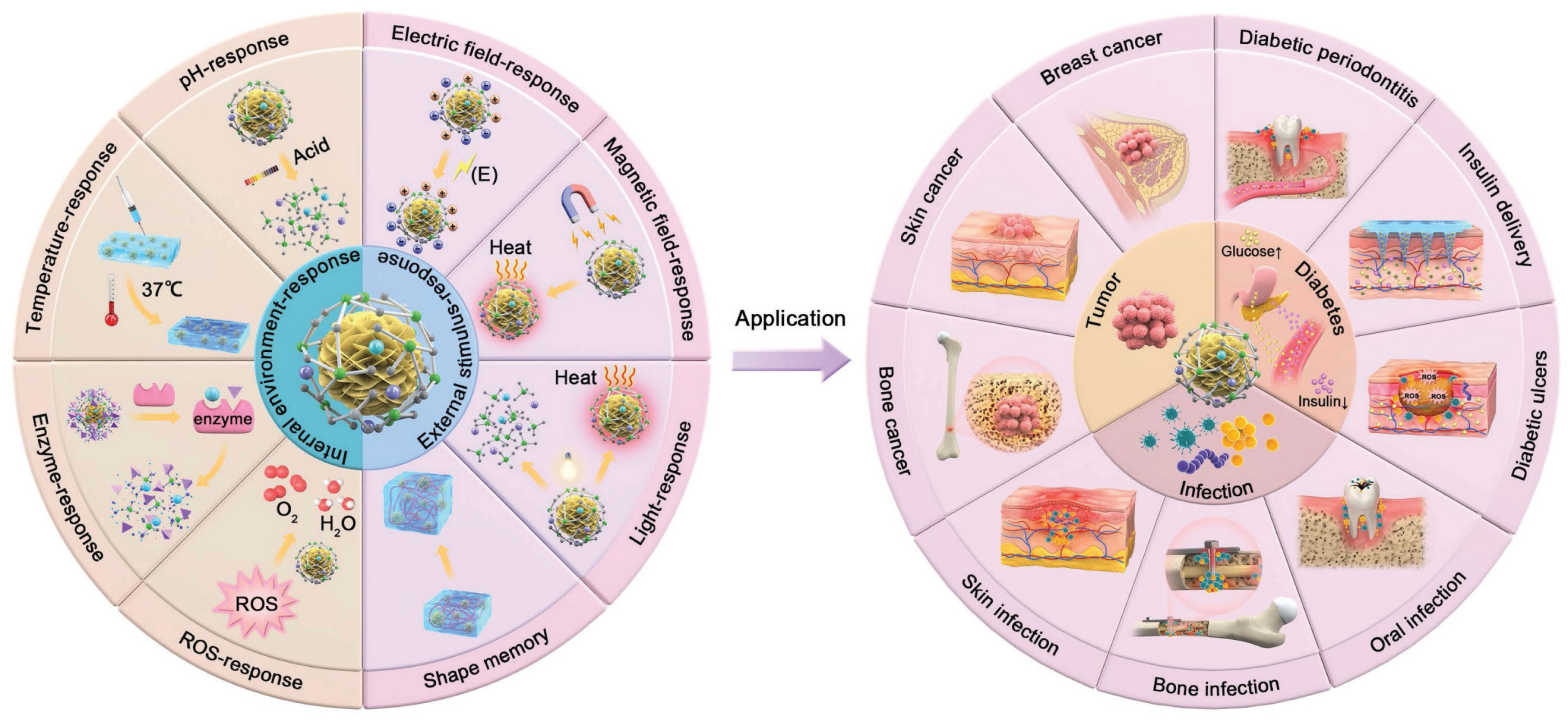
Schematic diagram of classification of SBGC and application in complex tissue defect repair.

Therefore, this review will provide an overview of the response types of SBGC in various internal and external environments, as well as their applications in multiple fields. At the same time, the current situation of SBGC is analyzed and their future development is predicted (Fig. 1).

## Synthesis method of SBGC

The preparation of SBGC is to add ions, drugs and compounds with smart response functions to the BG composite material. According to the location of the smart response function about the BG particles, it can be divided into the inside, the surface, and the outside of the BG particle.

SBGCs with smart response points located inside the BG particles were mainly prepared by ion doping. The -Si-O- network structure of BG allows the internal incorporation of various ions during the sol-gel synthesis process^6^. This capability has led to the development of various ion-doped BGs that exhibit intelligent response properties. Recently reported dopant ions include manganese, cerium, and bismuth, among others, conferring a smart response to the BG^7^-^9^. This controlled degradation facilitates sustained and effective stimulus responses, enhancing the material's suitability for therapeutic applications that require continuous ion release.

SBGCs with smart response points on the BG surface were mainly constructed by physical adsorption and chemical grafting. Electrostatic attraction is a common method for loading smart response points by physical adsorption. Mesoporous bioactive glasses (MBG) with very high specific surface area are often used as carriers for therapeutic ions. The mesopore structure is an advantage of the template method of preparing BG which can modulate the mesopore size by controlling the experimental conditions^10^. Due to the abundance of negatively charged silicon hydroxyl groups on the BG surface, positively charged drugs and ions can easily bind to the BG surface by electrostatic attraction^11^-^14^. In addition, smart response functional groups can also be chemically grafted onto the surface of BGs, including peptides, enzymes, *etc.*^15^-^17^. It is also possible to graft functional groups with high drug loading efficiency onto the surface of BG, and then graft smart responsive drugs to improve stability. For example, amino-functionalized MBG are positively charged and help to load more negatively charged drugs^18^.

SBGCs with smart response points outside the BG mainly form composite materials by combining them with other polymers such as collagen, sodium alginate, chitosan, ethylene glycol, and so on^19^,^20^. Smart response components can be polymers or drugs loaded into polymers. Based on the advantages of excellent suspension and uniform particle size, BG particles can be evenly mixed with organic polymers to produce electrostatic films, injectable hydrogels, 3D printing scaffolds,* etc.*^21^-^23^.

## Internal microenvironment-responsive SBGC

The internal microenvironment refers to the environment surrounding a cell in the body, including the chemical, physical, and mechanical conditions in which it is located and other cells and the extracellular matrix adjacent to it. The microenvironment influences the biological behavior of cells, such as their migration, proliferation, differentiation, and apoptosis^24^-^27^. Under disease conditions, some physicochemical parameters in the microenvironment, such as pH, reactive oxygen species (ROS) concentration, and temperature, undergo specific changes^28^-^31^. The SBGC specifically responds to changes in these parameters of the microenvironment and provides appropriate feedback, such as structural changes and drug release, leading to targeted treatment of diseases.

### pH-responsive SBGC

In disease states such as infections and tumors, high levels of cellular glycolysis and lactic acid accumulation cause a decrease in the pH of the microenvironment at the site of the lesion^32^-^35^. The SBGC responds to this acidic environment by releasing therapeutic drugs, achieving disease treatment, and promoting tissue regeneration. Physical adsorption and chemical grafting are common methods for constructing pH-responsive SBGC.

Physical adsorption combines pH-sensitive components with the porous structure of mesoporous bioactive glass. For example, ZnO quantum dots (ZnO QDs) are often used as "gatekeepers" for mesoporous structures because of their unique acid responsiveness^36^,^37^. ZnO QDs, which coat the pore structure of MBG through simple electrostatic interactions, remain stable at pH 7.4 but immediately dissolve into zinc ions at pH values less than 5.5^38^. At this point, the loaded drug is released to achieve specific treatment of the lesion. Another study took full advantage of the biological activity of BG to spontaneously mineralize through physical sedimentation, forming a hydroxyapatite (HAP) coating in the drug-carrying MBG pores, thereby limiting drug release. The gradual degradation of HAP in acidic environments and an ingenious realization of the pH response could then be utilized to control drug release^39^. In addition to the use of acid-sensitive materials to encapsulate drugs within MBG, some drugs themselves can be physically adsorbed onto the MBG surface^40^. For example, the amino groups in the antitumor drug doxorubicin (DOX) are readily protonated in acidic media to become positively charged NH^3+^, which allows DOX to desorb from the MBG surface by electrostatic effects^41^. Thus, DOX-loaded MBG has pH-sensitive drug release capabilities. In addition, the drug chelates with Ca^2+^ on the MBG and is released when Ca^2+^ is released from the MBG nanospheres. The ionic dissolution characteristics of BG provide a new idea for the design of pH-responsive SBGC^42^. The construction of a pH-responsive SBGC system by physical adsorption is a relatively simple method. However, considering the complex and changeable cellular microenvironment, its stability must be further studied.

In addition to physical methods, chemical grafting is also an effective method for constructing pH-responsive SBGC. Chemical grafting introduces acid-sensitive functional groups onto the MBG surface. Various anhydrides and polypeptides are common acid-sensitive functional groups. Under acidic conditions, the covalent bonds of these functional groups are broken for the controlled release of therapeutic drugs^43^-^45^. For example, poly-L-glutamic acid (PLGA) is a synthetic peptide that has been extensively studied for its pH response due to its modifiable carboxyl side groups^46^,^47^. Amino-silane-functionalized MBG is covalently linked to PLGA via an amide bond to construct a pH-responsive SBGC for the delivery of daunorubicin. At a pH value of approximately 5.5, daunorubicin is significantly released^48^.

However, tissue regeneration is a dynamic process. For example, the initial local environment of a fracture hematoma is acidic, gradually becoming neutral and eventually alkaline as healing progresses^49^. Therefore, real-time monitoring of pH at the lesion site helps to provide timely feedback on disease status. Aggregation-induced emission luminogens (AIEgens) have attracted much attention because of their high luminous intensity in the aggregation state^50^. AIEgens are attached to MBG by post-grafting. The emission wavelength of the SBGC reversibly changes with changing pH, which is used as an effective fluorescent probe for cell imaging. Moreover, SBGC, which has high drug loading efficiency, enables sustained, pH-responsive drug release^51^,^52^. In addition, the SBGC should be able to implement sequential responses based on changes in pH. In an acidic environment, one therapeutic drug is released to reduce the inflammatory response and treat the associated disease, whereas, in an alkaline environment, another drug is released to promote tissue repair. This dual drug delivery strategy enables precise drug release in different disease states and simplifies drug delivery procedures^53^.

pH-responsive SBGC has a wide range of applications in the field of tissue regeneration. However, MBG generally has high degradability in acidic environments. The release of calcium and phosphorus ions may lead to a sudden increase in local pH, which is not conducive to the precise release of drugs and may cause damage to normal cells^54^,^55^. To solve this problem measures such as changing the structure, composition, or assembly method of SBGC can be taken to adjust its solubility, thereby reducing drastic changes in pH. In addition, future research directions can focus on the development of systems for the timely monitoring of pH changes and drug release in the application area.

### ROS-responsive SBGC

ROS are highly active oxygen compounds produced by cell metabolism. By mediating redox signaling, ROS maintain the intracellular redox state and regulate cellular function^56^. However, in the disease state, an imbalance in redox homeostasis leads to the overproduction of ROS, which leads to oxidative stress and ultimately induces inflammation and pathological responses^57^,^58^. SBGC utilizes polyphenols and antioxidant metal ions to relieve oxidative stress in response to excess ROS at the lesion site.

**Figure 2:**
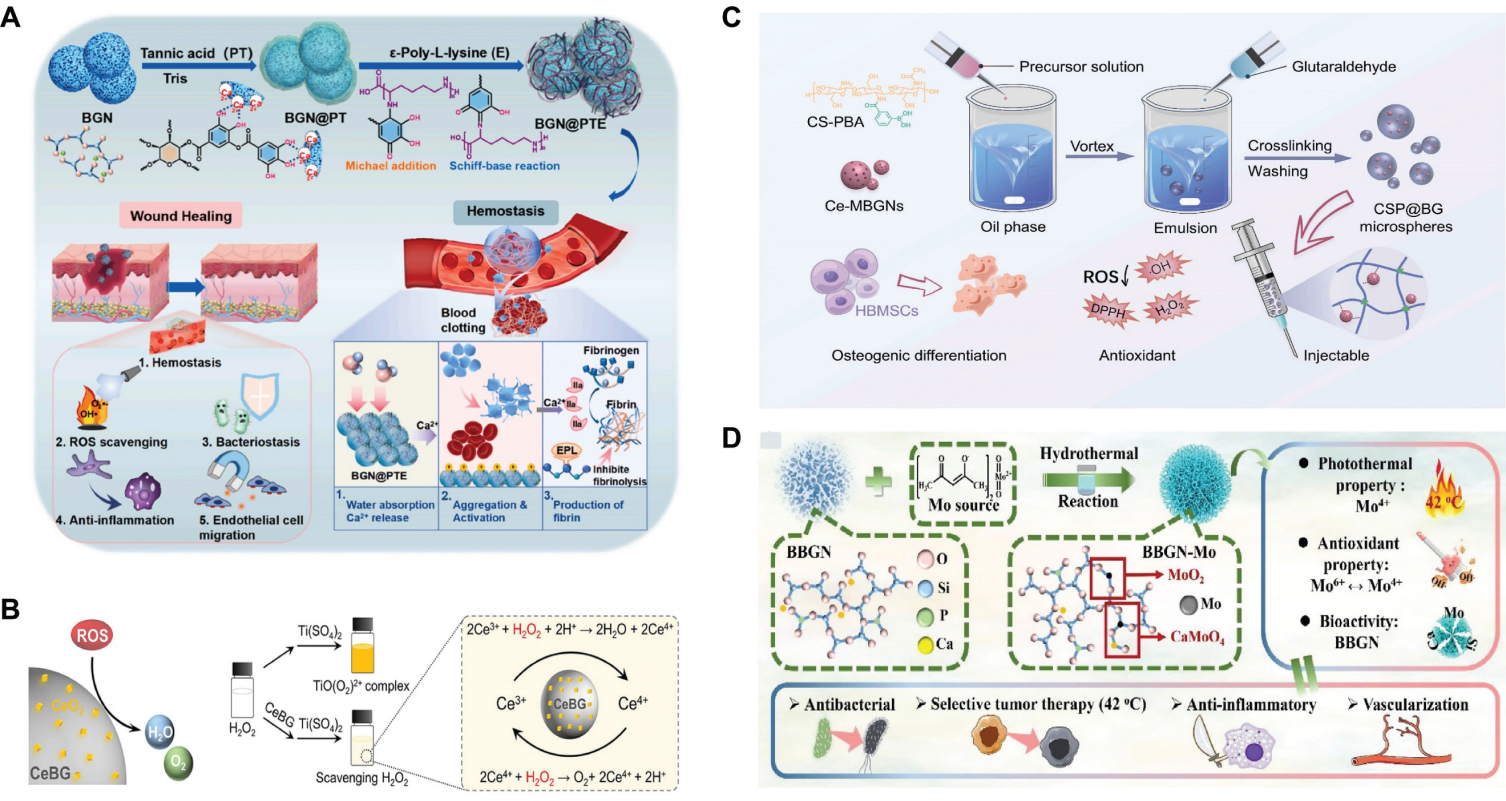
** ROS-responsive SBGC:** (A) Description of the synthesis route of multi-layer BGN@PTA nanosystems. Adapted with permission from 65, copyright 2023 Elsevier. (B) Antioxidative mechanism diagram of Ce-BG. Adapted with permission from 66, copyright 2024 Elsevier. (C) Instructions for the preparation of injectable organic/inorganic composite CSP@BG-microsphere systems. Adapted with permission from 69, copyright 2023 Elsevier. (D) Synthesis and structural analysis of B-M with multifunctional activity. Adapted with permission from 71, copyright 2021 ACS publications.

First, the grafting of polyphenols is a method to implement SBGC responses to ROS. Polyphenols have complex polyphenol structures, including benzene rings, hydroxyl groups, aldehyde groups, and other functional groups. These functional groups react with free radicals and exhibit antioxidant activity^59^,^60^. However, these chemical structures also mean that polyphenols are unstable and easily degraded, causing them to lose their antioxidant activity^61^. Polyphenols conjugated to BG improve their molecular stability and bioavailability to a certain extent^62^,^63^. For example, polytannic acid (PTA) and ε-polylysine (ε-PL) functionalize BG through layer-upon-layer assembly. The coordination interactions between the calcium ions in BG and PTA result in stable covalent bonds (Fig. 2A). The active hydroxyl group of PTA responds to high levels of ROS by donating hydrogen atoms to free radicals^64^,^65^.

Second, SBGC doped with various valence ions also actively responds to ROS. Cerium(Ce)-doped SBGC responds to and quenches ROS via a transition of oxidation states between Ce^4+^ and Ce^3+^(Fig. 2B)^66^. Notably, the ratio of Ce^3+^/Ce^4+^ affected the type of ROS catalysis. High Ce^3+^/Ce^4+^ ratios resulted in significant superoxide dismutase mimetic activity, whereas low Ce^3+^/Ce^4+^ ratios resulted in significant catalase (CAT) mimetic activity^67^,^68^. Therefore, the ratio of Ce^3+^/Ce^4+^ in the SBGC can be adjusted as needed. In further studies, injectable SBGC microspheres were developed by combining Ce-BG with chitosan (CS) using water-in-oil emulsification. The phenylboric acid group of CS endows SBGC with excellent tissue adhesion properties, whereas Ce-BG responds to and scavenges various reactive oxygen species such as hydrogen peroxide (H_2_O_2_) and ⋅OH, effectively alleviating oxidative stress (Fig. 2C)^69^. In addition to Ce, molybdenum (Mo) species, mainly Mo^6+^ and Mo^4+^ are also typical variable valence elements^70^. Mo^4+^ and Mo^6+^ can be added to the BG network using a hydrothermal-assisted classical synthesis method. This SBGC structure has many free electrons and oxygen vacancies, which are the main factors in the response to ROS. In addition, the stabilized free electron and oxygen vacancies endow SBGC with long-term effective antioxidant activity and photothermal properties (Fig. 2D)^71^.

To obtain SBGC with better ROS responsiveness, simultaneous loading of Ce and polyphenols has also been investigated^72^. Notably, however, moderate levels of ROS play a key role in regulating cellular metabolism and the stress response, which is conducive to maintaining cell and tissue health^73^. Current research on ROS-responsive SBGC focuses on how to maximize the response to and scavenging of ROS; however, the monitoring and regulation of ROS levels are still lacking. Therefore, it is possible to design an SBGC capable of maintaining stability over a specific range of ROS concentrations, which implies a more sensitive response of SBGC to ROS.

### Temperature-responsive SBGC

The normal body temperature is usually between 36.5 °C and 37.5 °C. Within this temperature range, the physiological functions of the body can function normally^74^. The temperature-responsive SBGC uses human body temperature to trigger response behavior. When the SBGC experiences a change from ambient temperature to the physiological temperature of the human body, its shape and structure change.

The combination of BG and a temperature-sensitive hydrogel is a common method to construct a temperature-responsive SBGC. A temperature-responsive SBGC has the advantages of being injectable and undergoing a sol-gel transition at body temperature to form a stable structure^75^. Based on the source of raw materials, hydrogels are categorized into synthetic hydrogels and natural hydrogels. The p(N-isopropylacrylamide-co-butyl methylacrylate) (PIB) nanogels are synthetic crosslinked polymers. The dispersions show three phase states at different temperatures, including expansive gel, mobile sol, and shrink gel. For example, boron-containing MBG in combination with a PIB nanogel forms a temperature-responsive SBGC that undergoes rapid thermal gelation at body temperature and is suitable for the repair of irregularly shaped bone defects^76^.

In addition, hydrogels obtained by physical cross-linking of natural polysaccharides also respond to temperature^77^. The SBGC constructed with a combination of Cu-BG and CS/silk fibroin/sodium glycerophosphate hydrogels is capable of in situ gel formation triggered by body temperature and shows sustained and controlled release of Si, Ca, and Cu ions^78^,^79^. A similar study combined CS and gelatin polymers with BG nanoparticles to synthesize novel in situ-formed SBGC hydrogels. The incorporation of gelatin and BG significantly improved the elastic properties of SBGC and effectively shortened the gelation time^80^.

Fever has long been considered one of the indicators of the acute phase of infection or injury^81^,^82^. Current research on temperature-responsive SBGC has focused on the physical conformational changes that occur in hydrogels from room temperature to body temperature. However, this approach does not allow for a more precise and flexible temperature response. Responding to subtle changes in body temperature during disease states may be an interesting research direction for temperature-responsive SBGC.

### Enzyme-responsive SBGC

Enzyme-responsive SBGC utilizes specific binding between the enzyme and the substrate^83^. The SBGC carries substrates that respond to enzymes that are abnormally expressed at the lesion site, triggering a catalytic reaction. As the substrate is consumed, the SBGC undergoes structural changes in which the encapsulated drug is released.

Enzyme-responsive SBGC is constructed by grafting an enzyme substrate onto MBG. The substrate acts as a molecular gate covering the mesoporous structure of MBG. When the enzyme whose expression is abnormally high at the lesion site reacts with the substrate, the molecular gate unblocks the mesoporous structure. For example, the high-energy phosphate compound adenosine triphosphate (ATP) is a commonly used energy carrier in cells and is used as a substrate for building an SBGC against acid phosphatase (ACP), which is highly expressed at sites of bone infection. ATP forms covalent bonds with triamines on the outer surface of MBG to cover the pores and inhibit the release of antibiotics. The increased ACP at the infected bone tissue site causes hydrolysis of the phosphate bond of ATP, opening the entrance of the SBGC mesopore and releasing antibiotics^84^. In similar studies, high levels of alkaline phosphatase (ALP) in the serum of patients with osteosarcoma have been used as a target for an enzyme-responsive SBGC. ε-PL was shown to function as a molecular gate closure functionalized for MBG to enable targeted drug release at tumor sites in the presence of ALP^85^.

Enzyme-responsive SBGC is a less-studied area that needs to be further explored. Currently, the most common therapies for the enzyme response include self-assembly of peptides with enzyme cleavage sequences or covalent attachment of protease-sensitive peptides to biomaterials^86^,^87^. However, these methods have not been applied to construct an enzyme-responsive SBGC. In addition, some enzymes are distributed in multiple cells, such as glucokinase, which is widely distributed in the cytoplasm^88^. Nonspecific catalytic reactions inevitably lead to unintended side effects. In this case, membrane camouflage can provide a solution^89^. Membrane-camouflaged SBGC selectively enters specific cells to catalyze reactions with intracellular enzymes, which may be an interesting research direction.

In brief, we have summarized how SBGCs specifically respond to pH, ROS, temperature, and enzymes in the pathological microenvironment, providing new insights into the complex interactions between biomaterials and their cellular environment (Table 1). The cellular microenvironment is a complex, dynamic, three-dimensional environment. SBGCs modulate the cellular microenvironment and modulate its structure or function in response to changes in physical or chemical parameters surrounding the cell. At present, these types of SBGC are still in the preliminary stage of research, and further investigation is needed to optimize their composition and structure, as well as their interaction mechanisms with the cell surface. This includes how SBGCs affect key biological processes, such as cell signaling, gene expression, and the cell cycle, and precisely regulate cell proliferation, differentiation, and function. In addition, other parameters in the disease microenvironment, such as potential and ion concentrations, are worthy of further study.

## External stimulus-responsive SBGC

An exogenous stimulus-responsive SBGC means that external stimuli change the structure, nature, or function of the cell to achieve specific treatment of diseases^90^-^92^. Common external stimuli include electric field, light, and magnetic field (Table 2).

### Electrical stimulus-responsive SBGC

Electrical stimulation (ES) is a physical therapy that promotes tissue healing and functional recovery through external electrical stimulation^93^. However, ES is not spatially limited and is less effective at repairing deep tissue. An electrical stimulus-responsive SBGC compensates for this shortcoming by responding to external electrical signals and delivering electrical stimulation to the target tissue while promoting tissue repair^94^,^95^.

Polarization and incorporation of conductive materials are effective methods for constructing an electrical stimulus-responsive SBGC. First, the polarization of the BG itself is a strategy for building a stimulus-responsive SBGC. High temperatures and voltages are applied to the BG to create a permanent electric dipole moment. Due to the migration of sodium ions, a large surface charge is stored in the BG, which changes the internal structure of the BG and results in a certain conductivity^96^,^97^. Polarization results in a change in the morphology of the BG silicon-rich layer. When external electrical stimulation is applied, the charge within the BG rearranges, triggering a specific bioactive response that regulates the growth rate of amorphous calcium phosphate and bone apatite^98^. Second, the BG is combined with highly conductive materials to form a conductive network. For example, carbon nanotubes (CNTs) coated on the surface of a BG scaffold by electrophoretic deposition respond to external electrical stimulation, which has the potential to increase cell viability and differentiation^99^. In addition, CNTs are used as sensors to monitor the bioactivity level of BG. CNTs form a conductive network in the BG matrix and use current-voltage characteristic changes as markers of biological activity to develop SBGCs with sensing functions^100^.

**Figure 3:**
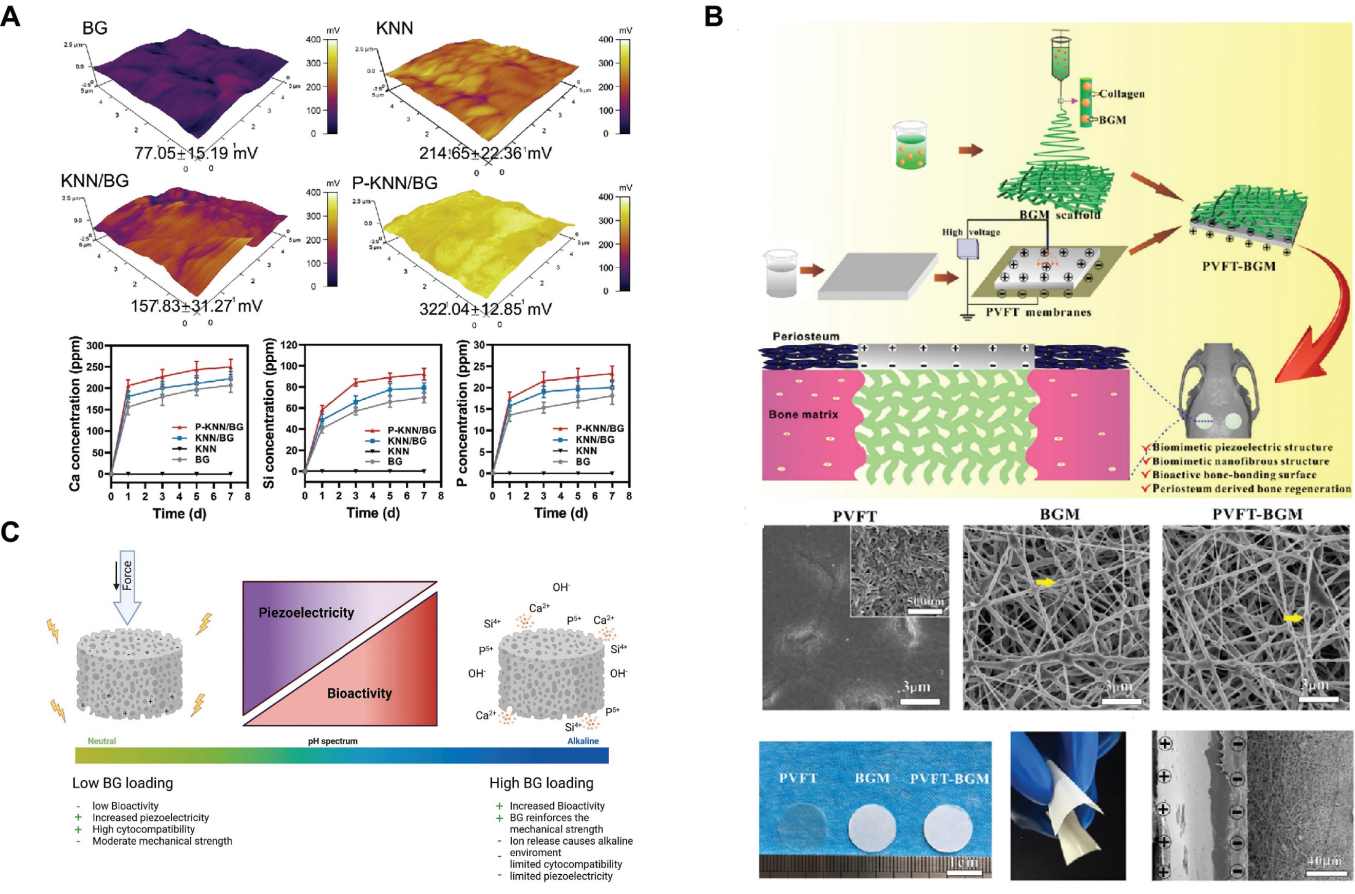
**Electrical Stimulus-responsive SBGC:** (A) Surface potential and ion release profiles of KNN / BG and P - KNN / BG. Adapted with permission from 102, copyright 2023 Wiley-VCH. (B) Preparation and observation of PVFT - BGM composite scaffolds. Adapted with permission from 103, copyright 2020 Elsevier. (C) Influence of BG loading on the basic properties of 3D-printed BaTiO_3_ / BG composites. Adapted with permission from 104, copyright 2023 Elsevier.

Some limitations remain in the process of electrical stimulus-response, such as the need for an external power supply to power and regulate the stimulus conditions of the exogenous electric field. Notably, piezoelectric materials provide a solution for generating charge distribution changes without the need for an external power source^101^. Piezoelectric materials promote bone regeneration by accumulating electrical charge in response to mechanical stress. For example, potassium sodium niobate (KNN) is a lead-free piezoelectric material. The KNN-doped SBGC (KNN/BG) is prepared via a solid-phase synthesis route. Subsequently, an external electronic device polarizes the KNN/BG to obtain P-KNN/BG, whose regular domain arrangement will help to improve the piezoelectric properties (Fig. 3A). The SBGC responds to mechanical energy to form a stable electrical stimulus that induces hyperpolarization of the cell membrane, increasing the inward flow of active ions into the cell. The enhancement of endothelial cell adhesion, migration, and differentiation through the activation of the eNOS/NO signaling pathway facilitates angiogenesis^102^. In addition, our group developed a biomimetic bioactive piezoelectric SBGC that utilizes piezoelectric poly(vinylidene fluoride-trifluoroethylene) (PVFT) to mimic the periosteum and BG micro-nanoparticles to mimic the bone matrix (Fig. 3B). The PVFT gives full play to the electromechanical coupling effect, whereas the BG provides sufficient mineral ions (such as Ca and P) that are essential for bone formation^103^. However, the amount of BG loading is a factor to be considered. As shown in Fig. 3C, the BG content should be within a moderate range. High concentrations of BG limit the piezoelectric properties, thus eliminating the possibility of an electrical stimulation response^104^.

### Magnetic field-responsive SBGC

Magnetic materials react in a certain way to an external magnetic field^105^. By changing their physicochemical properties and structure in response to a magnetic field, a magnetic field-responsive SBGC quickly and controllably achieves magnetothermal effects and the controlled release of therapeutic ingredients.

The magnetic field response capability of an SBGC is realized by introducing a magnetic phase. Iron oxides (such as Fe_3_O_4_ and γ-Fe_2_O_3_) are common magnetic phases^106^,^107^. BG has controllable magnetothermal properties due to the addition of iron oxide^108^-^110^. For example, iron oxide nuclei (γ-Fe_2_O_3_) are encapsulated within a BG shell to synthesize superparamagnetic SBGC, which is used as a bone filler material after tumor resection. This SBGC is obtained in two steps: coprecipitation of 16 nm iron oxide nanoparticles followed by growth of a bioactive glass shell layer by a modified Stöber method^111^. In addition to magnetothermal effects, SBGC also controls ion release in response to magnetic fields. High-porosity magnetic bioactive glass is prepared by the sol-gel method and then combined with calcium sulfate to form the SBGC. This SBGC releases Ca^2+^ in response to an *in vitro* magnetic field, leading to calcium overload and death of tumor cells while producing a magnetothermal effect^112^.

However, the bioactivity of BG decreases with increasing Fe concentration because the Fe content affects the nucleation and growth of apatite on the BG surface^113^; this may be due to the reaction of Fe in iron oxide with the Si, P, and Ca in the BG to form nonmagnetic phases, such as Fe_2_(SiO_3_)_3_ and FePO_4_^114^. To solve this problem, various methods have been adopted. First, a new sol-gel route was developed to prepare a field-responsive SBGC. Heat treatment of iron-doped BG promoted the growth of α-Fe_2_O_3_ nanocrystals, which endowed BG with superparamagnetic properties sufficient to generate heat under an applied alternating magnetic field. Moreover, The crystallization did not inhibit the biological activity of BG^115^. Second, iron oxide was modified with graphite. The chemically stable graphite is adsorbed on the surface of the iron oxide and remains between the iron oxide and the glass matrix, preventing the iron ions from forming a nonmagnetic phase. The SBGC formed by this method has considerable biological activity and a better magnetothermal response^116^. Finally, the iron oxide is replaced by Ba ferrite (BaFe_12_O_19_, BF) to form a magnetic field-response SBGC whose main phases are calcium sodium silicate and barium iron oxide. The magnetic field responsiveness and biological activity increased with increasing BF content^117^.

In terms of drug delivery, the timed and quantitative release of drugs is achieved by adjusting the intensity and frequency of the external magnetic field. This smart approach compensates for the shortcomings of traditional drug delivery, ensuring that the concentration of the drug in the body remains within the therapeutic range at all times^118^. No studies have focused on magnetic field-responsive SBGC for the controlled release of smart drugs. In addition, the magnetic field response ability of SBGC is combined with magnetic hyperthermia, chemotherapy, chemodynamic therapy, and other methods to improve the efficacy of tumor treatment^119^-^122^.

### Light-responsive SBGC

The light response refers to the controlled and reversible change in a smart material after light irradiation^123^,^124^. The light response properties of SBGC are achieved by introducing specific light-sensitive components. After specific wavelengths of light are absorbed, certain chemical or physical changes, such as photothermal effects, drug release, and shape changes, occur within or between molecules, exhibiting specific functions^125^,^126^. The light-sensitive components introduced in SBGCs mainly include inorganic nanomaterials and organic compounds.

**Figure 4:**
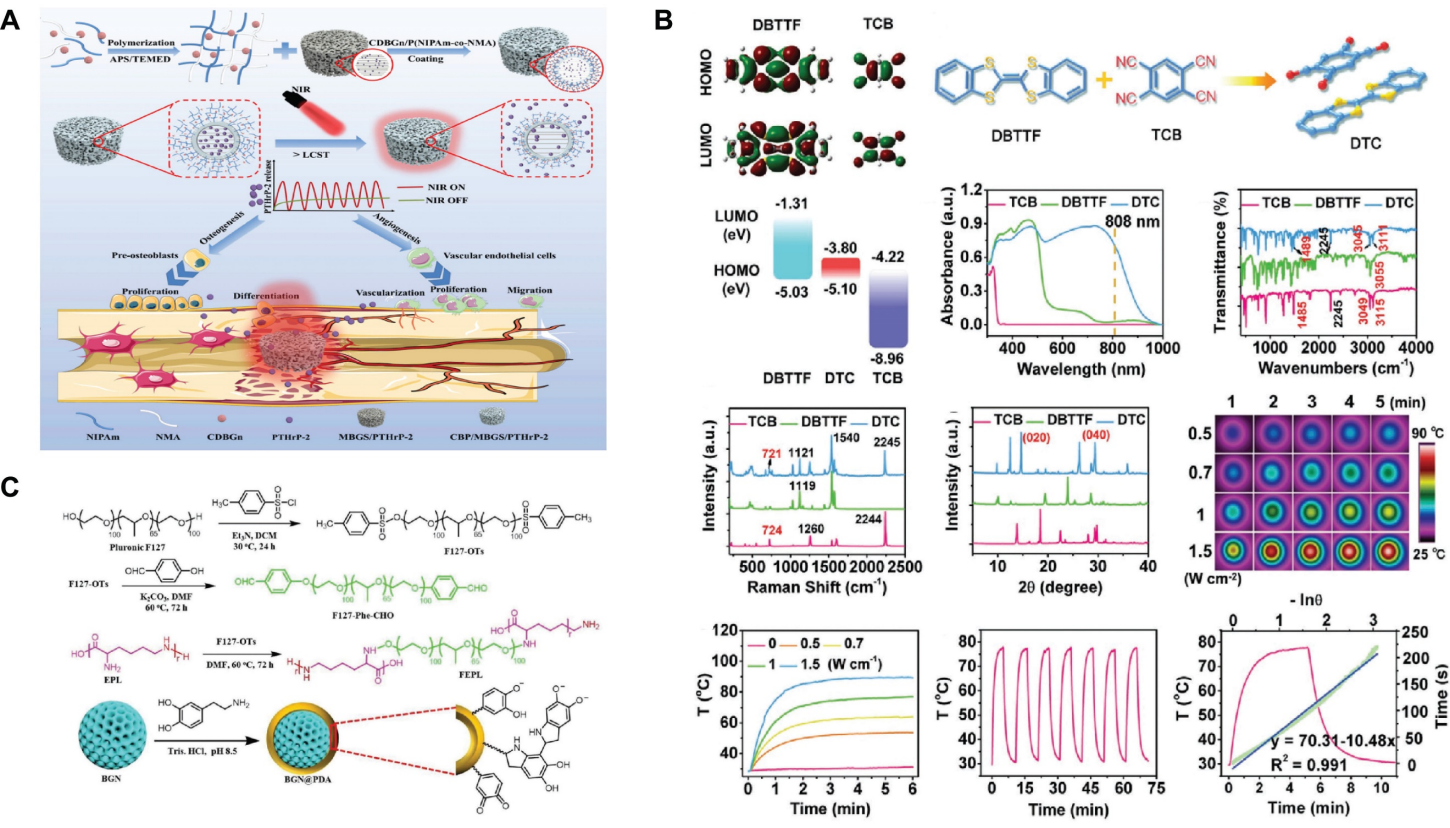
** Light-responsive SBGC:** (A) LCST is a switch to control drug release by being turned on or off during NIR irradiation. Adapted with permission from 136, copyright 2023 Elsevier. (B) Characterization and photothermal performance of DTC cocrystal. Adapted with permission from 140, copyright 2020 ResearchGate. (C) PDA-functionalized BG nanoparticles and F127-ε-polylysine formed a hydrogel network via Schiff base reaction. Adapted with permission from 144, copyright 2021 Wiley-VCH.

Certain inorganic nanomaterials, most notably metal nanoparticles, have been recognized as excellent reagents for light response, as have carbon-based nanomaterials and semiconductor nanoparticles^127^. First, the free electrons of the metal nanoparticles interact with the light field to produce the surface plasmon resonance (SPR) effect, which effectively absorbs and scatters light at specific wavelengths and converts the light energy into thermal energy^128^,^129^. Fe-, Mn-, Cu-, Mo-, and Bi-doped SBGCs show photothermal properties, indicating that they can be used for the photothermal treatment of tumors and infections. The final temperature of the SBGCs is controlled by varying the type and content of the doped metal and the laser power density^71^,^130^-^133^. In particular, Bi-doped SBGC effectively controls photothermal effects by quenching luminescence or depolymerizing glass networks^127^. In addition, nanoparticles modified with peptides can be used as molecular gated systems. The MBG loaded with simvastatin was terminated with gold nanoparticles modified with E7 peptide, and controlled release of simvastatin was achieved after NIR irradiation^134^. Second, carbon nanomaterials are also light-responsive. Two-dimensional ultrathin niobium carbide MXene nanosheets can be incorporated into 3D-printed BG scaffolds (NBGS) for treating osteosarcoma. NBGS has superior light-responsive properties in the near-infrared-II (NIR-II) biological window, allowing for deeper tissue penetration^135^. A similar study has incorporated carbon-doped BG nanoparticles into polymer hydrogels as photothermal converters for the delivery of parathyroid hormone. As shown in Fig. 4A, the lower critical dissolution temperature (LCST), as a switch to control drug release, is turned on or off during NIR irradiation^136^. Finally, Cupr-based chalcogenides, as semiconductors, constitute a new class of highly efficient photothermal reagents. CuFeSe_2_ nanocrystals are grown in situ on the surfaces of BG scaffolds during the solvothermal reaction. Under 808 nm NIR irradiation, the photothermal response of SBGC scaffolds can be adjusted by changing the CuFeSe_2_ nanocrystalline content and laser power density^137^; this provides a way to further develop light-responsive SBGCs. It is well known that BG contains a large amount of silicon, which is an excellent semiconductor material widely used in the manufacture of electronic devices^138^. Therefore, synthesizing light-responsive BG without relying on photosensitizers might be a challenging research direction.

The SBGC combined with organic compounds also shows light responsiveness. For example, coumarin groups are grafted onto MBG for a light-controlled molecular gate, effectively controlling the light response aperture. UV light (> 310 nm) irradiation induces photodimerization of coumarin to close the pores with cyclobutane dimers. At this point, drug molecules cannot escape from the individual pores of the MBG. However, irradiation with shorter wavelengths of UV light (250 nm) cleaves the coumarin dimer regenerating the coumarin monomer, opening the pore and allowing the drug molecule to be released^139^. In addition to the use of grafted organic compounds, the cocrystal strategy is also an effective method to impart BG scaffolds with light-responsive properties. A bifunctional SBGC scaffold (DTC@BG) is engineered by the in-situ growth of NIR-absorbing DTC cocrystals on the surface of a 3D-printed BG scaffold. Two small molecules, dibenzotrathiafulvalene as the electron donor and tetracyanobenzene as the electron acceptor are selected to prepare DTC cocrystals, which exhibit excellent photothermal conversion performance due to the intense charge transfer interaction between the acceptor and donor units (Fig. 4B)^140^. Finally, recent studies have shown that the introduction of substances such as hematin and polydopamine (PDA) into BG scaffolds also enhances their photothermal effects^141^,^142^. The light responsiveness of hematin may be derived from the iron ions in its molecular structure. PDA is an efficient photothermal conversion material and has the advantages of strong adhesion, easy preparation, and modification. The abundance of functional groups (*e.g.*, catechol, amines, and imines) in PDA alters the surface properties of the BG^143^. PDA-functionalized BG is readily attached to other biopolymers by amidation with PDA or Schiff base reactions. As shown in Fig. 4C, PDA-functionalized BG nanoparticles, and F127-ε-polylysine form a hydrogel network via a Schiff base reaction, which exhibits excellent photothermal performance under NIR irradiation^144^.

In the process of photothermal therapy, the development of an SBGC with temperature monitoring and tissue repair functions is important. In recent years, rare earth ions (*e.g.*, Eu^3+^ and Nd^3+^) have been widely used in the fields of bioimaging, biosensing, and self-monitoring therapy due to their excellent luminescence properties^145^-^147^. Nd-Ca-Si bioactive glass, which is a new kind of multifunctional smart material with photothermal functions, fluorescence temperature measurements, and bioactivity, is prepared using a containerless processing technique. Due to the linear correlation between fluorescence intensity and temperature, this SBGC is used in photothermal therapy for in situ temperature measurements at tumor sites^148^. This provides a new possibility for the construction of light-responsive SBGC.

### Shape memory SBGC

The most remarkable feature of shape memory materials is their ability to restore their original shape in response to a stimulus, including heat, stress/pressure, current/voltage, magnetic field, pH change, solvent/moisture, and light^149^,^150^. Shape memory SBGC combines shape memory characteristics with the BG's biological activity and is perfectly adapted to the size and shape of the tissue site to be regenerated.

The main methods for constructing shape memory SBGC include combining BG with artificial and natural polymers with shape memory functions. For example, electrospun fibers of shape memory triethoxysilane-terminated poly(epsilon-caprolactone) (PCL-TES) loaded with BG are produced. The triethoxysilanyl group in the polymer is hydrolyzed and condensed with the silicon hydroxyl group on the surface of the BG particles, forming a superjunction compared with the PCL-TES system. Finally, PCL-TES/BG fibers showed excellent shape memory performance in terms of shape fixation rate and shape recovery rate^151^.

**Figure 5:**
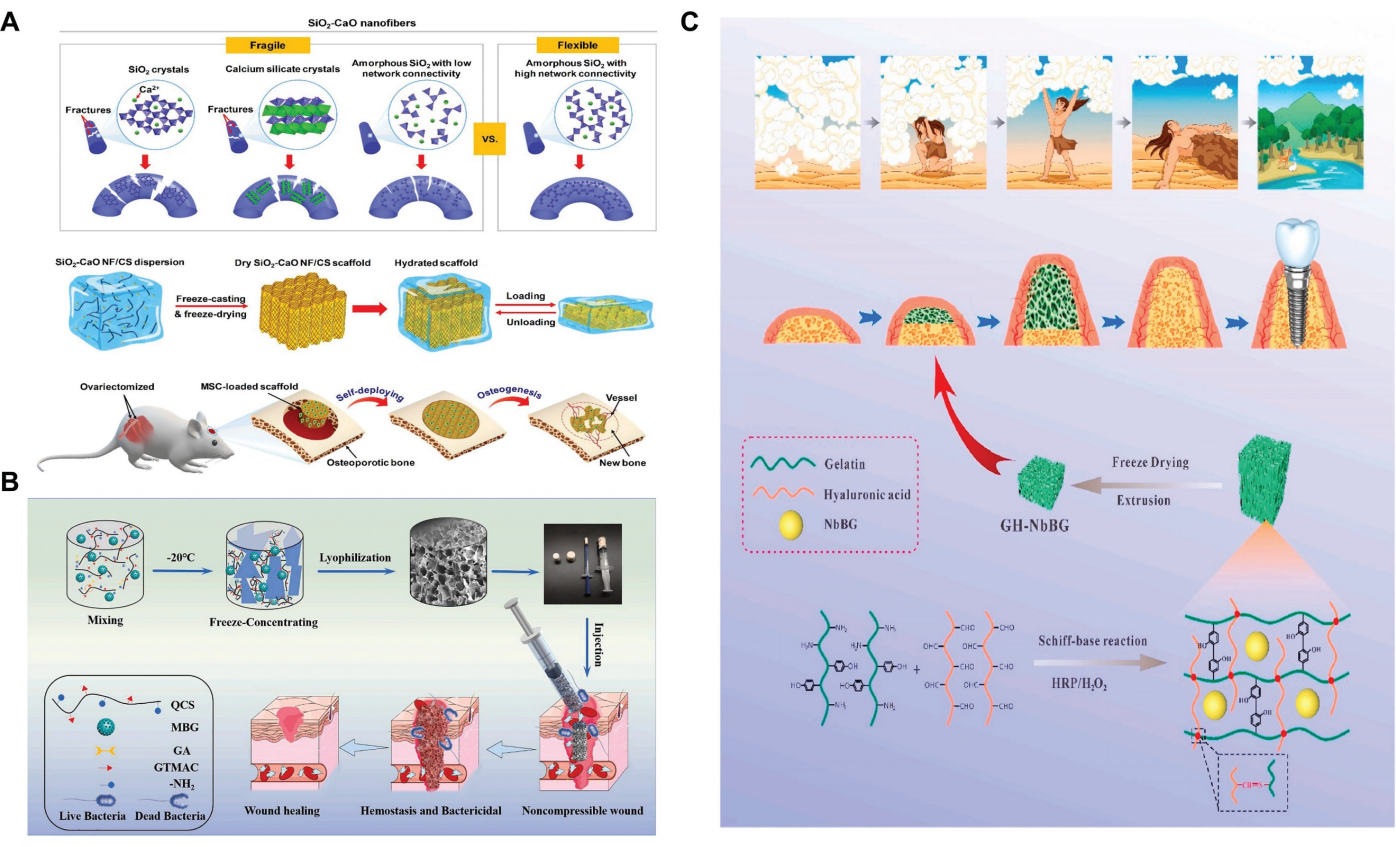
** Shape memory SBGC:** (A) Deformation and adaptation of irregularly shaped bone defects by elastic SBGC scaffolds. Adapted with permission from 154, copyright 2019 ACS Publications. (B) QCS-BG preparation method and its application as a crystalline gel. Adapted with permission from 155, copyright 2022 Elsevier. (C) Schematic diagram of GH-Nb BG hydrogel. Adapted with permission from 156, copyright 2023 Elsevier.

Another way for BG to realize shape memory function is to combine it with natural polymers. Natural polymers such as chitosan can trigger a shape memory function through hydration^152^. The shape-responsive SBGC constructed using this method fully utilizes the shape memory ability of chitosan and the induced mineralization advantage of BG^153^. For example, BG nanofibers with excellent flexibility and bioactivity have been successfully developed by manipulating their crystallization and chain configuration. Using chitosan as a linker, the BG nanofibers are further assembled into a 3D fibrous scaffold that exhibits elastic behavior with full recovery from 80% compression. As shown in Fig. 5A, the elastic fiber scaffold deforms and adapts to irregularly shaped bone defects and subsequently undergoes a self-deployment behavior to achieve a perfect match with the defects^154^. The water absorption and expansion properties of chitosan result in excellent hemostatic ability. Quaternized chitosan (QCS) is mixed with MBG and frozen to promote cross-linking to prepare a multifunctional shape memory cryo-hydrogel. It is capable of rapidly absorbing blood and regaining its shape upon compression, ultimately forming a physical barrier to block sites of bleeding (Fig. 5B). Positively charged functional groups on the QCS molecular chain form an adhesion barrier by physically cross-linking blood cells. Moreover, MBG activates the intrinsic coagulation cascade through its surface negative potential, releasing bioactive ions to promote wound healing^155^. However, chitosan is not the only natural polymer with shape memory. Our research team selected a double cross-linked gelatin-hyaluronic acid hydrogel with self-expanding properties and successfully dispersed niobium-doped bioactive glass into the hydrogel network (Fig. 5C). In this study, the disadvantage of hydrogel self-expansion was the advantage of a shape memory response to achieve self-expansion bone increment^156^.

Shape memory SBGSs show great potential in bone tissue engineering. Nevertheless, the current materials have several limitations, including low mechanical strength to cope with the complex biomechanical environment of bone defects. 4D printing technology is emerging as the next generation of printing technology^157^. The preparation of shape-memory BG scaffolds by 4D printing may exploit the shape-memory properties of bioactive glass itself.

## Applications of SBGC in complex tissue defects

### Applications of SBGC in anti-tumor

Tumors are pathological tissues formed by the abnormal proliferation of cells, for which surgical removal is the primary treatment. However, removing residual tumor cells and repairing defective tissues are still major challenges^160^. Although the regenerative effects of BG on hard and soft tissues have been confirmed, the ability to remove residual tumor cells is limited^161^,^162^. SBGC targets and eliminates residual tumor cells by intelligently responding to internal environments and external stimuli while remodeling defective tissues. This section summarizes the application of SBGC in three areas: bone tumors, skin cancer, and breast cancer (Table 3).

#### Bone tumor

For bone tumors, especially osteosarcoma, surgical resection cannot completely remove the tumor cells. Moreover, bone defects removed by tumors cannot be repaired by themselves^163^. While filling bone defects, SBGC eliminates residual tumor cells in response to the bone tumor microenvironment and external stimuli. Advanced methods such as local chemotherapy, bioimaging, chemodynamic therapy (CDT), photodynamic therapy (PDT), and hyperthermia are realized due to the responsiveness of SBGC.

First, SBGC achieves targeted release of anti-tumor drugs and bioimaging by responding to the internal and external environment. On the one hand, the acidic microenvironment of the tumor acts as the target of SBGC's response to achieve the release of anti-tumor drugs^164^. For example, DOX, a commonly used tumor chemotherapeutic, was loaded into functionalized MBG and targeted for release in the acidic microenvironment of bone tumors, effectively inhibiting bone tumor growth. Meanwhile, MBG contributes to long-term bone tissue regeneration^42^,^165^,^166^. On the other hand, monitoring drug release behavior and biomineralization is critical for the treatment of deep bone tumors^167^. Upconversion nanoparticles (UCNPs) are widely used in bioimaging due to their ability to emit visible and NIR light under NIR excitation^168^,^169^. Among these, Er, Yb, *etc*. are often incorporated into the MBG networks to monitor biomineralization and DOX delivery behavior during bone tumor therapy^167^,^170^-^173^. For example, the results of biomineralization monitoring showed that the upconversion luminescence intensity of MBG:4Er/3Yb bursts with increasing biomineralization. Meanwhile, DOX quenched the green emission when loaded into MBG: 4Er/3Yb. When DOX was released, the green emissions recovered stably^167^. In another study, MBG/UCNPs (NaYF4: Yb/Er) were found to have intense red-light emission and could be used for drug delivery and drug delivery monitoring. In addition, MBG/UCNPs significantly enhanced the early osteogenic differentiation of bone mesenchymal stem cells (BMSCs) in bone tumor defects through the activation of osteogenic markers ALP, Runt-related transcription factor 2 (Runx2), osteopontin, osteocalcin and bone salivary protein^172^.

**Figure 6:**
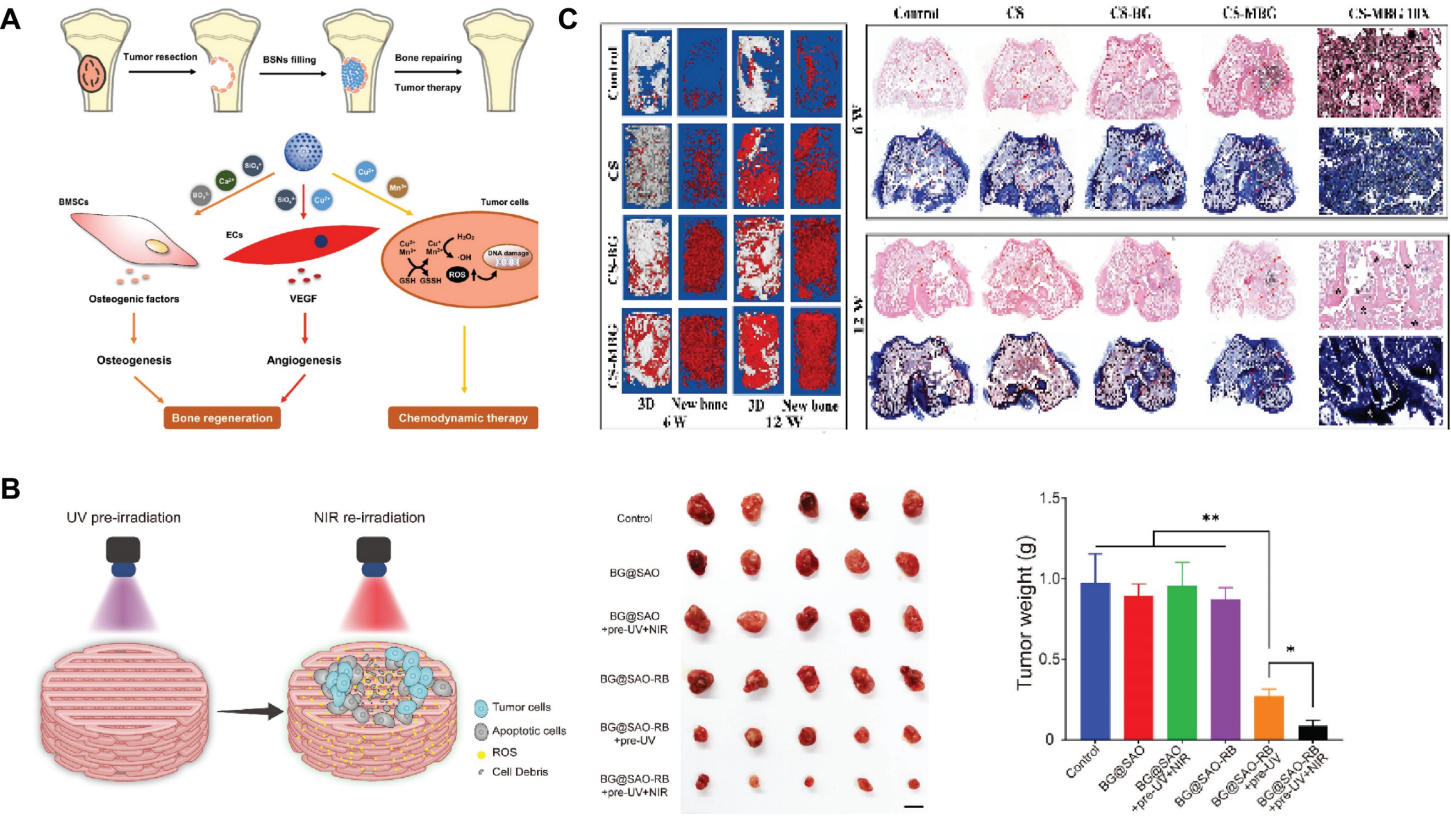
** SBGC in bone tumors:** (A) Schematic illustration of BSNs used for combined bone repair and anti-tumor. Adapted with permission from 176, copyright 2022 Elsevier. (B) *In vivo* osteosarcoma ablation efficacy of the BG@SAO-RB scaffold. Adapted with permission from 177, copyright 2024 Elsevier. (C) *In vivo* detection of osteogenesis by micro-CT and histopathological examination. Adapted with permission from 112, copyright 2023 Elsevier.

Secondly, SBGC produces toxic chemicals through CDT and PDT that trigger apoptosis of bone tumor cells^174^,^175^. For example, Cu^2+^ and Mn^3+^ respond to the bone tumor microenvironment through ionic valence changes to enable CDT. While CDT induces apoptosis of tumor cells, the ions released by SBGC promote osteogenic differentiation and angiogenesis, which have a good repair effect on the defect after bone tumor resection (Fig. 6A)^176^. Except for CDT, SBGC produces a large number of ROS to realize PDT in response to light stimulation, which induces apoptosis in tumor cells. For example, inside the SBGC scaffold, a persistent luminescent material (SrAl_2_O_4_: Eu, Dy) is used as a rechargeable internal light source. The material stores the energy produced by the excitation light and continues to emit light after the irradiation stops, achieving long-term effective progressive elimination of PDT osteosarcoma. The scaffold also directly promotes osteogenic differentiation of BMSCs and accelerates bone regeneration (Fig. 6B)^177^.

Finally, SBGC eliminates residual tumor cells through the high temperatures generated by thermotherapy. Tumor cells are poorly thermotolerant compared to normal cells. Therefore, thermotherapy can selectively kill tumor cells while protecting normal cells and tissues from damage^178^,^179^. Photothermal therapy (PTT) and magnetothermal therapy (MTT) are two methods of generating this therapeutic temperature. PTT is a treatment method that uses targeted recognition technology to gather near tumor tissue and convert light energy into heat energy under an external light source to kill cancer cells^130^. The light-responsive SBGC has the characteristics of photothermal conversion. For example, the Cu-doped SBGC composite hydrogels prepared by our research group inhibit tumor growth *in vivo* during the early implantation period, which stimulates the osteogenic differentiation of BMSCs by upregulating the expression of bone-related genes, significantly promoting new bone formation. The photothermal effect of this SBGC originates from the oxides formed by copper ions. The photothermal temperature can be controlled by Cu-BG concentration and NIR power density^180^. Similarly, another study used Mn to achieve controlled photothermal properties to promote bone regeneration while eliminating residual cancer cells^181^. Subsequently, SBGC produces a thermal effect in response to an alternating magnetic field to realize MTT^182^,^183^. Magnetic bioactive glass with high porosity by adding an appropriate proportion of Fe_3_O_4_ combined with calcium sulfate to form SBGC scaffolds. Under the magnetic field *in vitro*, the temperature of the scaffold can reach about 43°C, causing the apoptosis of bone tumor cells. Meanwhile, Ca^2+^ released by scaffolds promoted the proliferation and differentiation of BMSCs and achieved the repair of bone defects (Fig. 6C)^112^.

PTT and PDT summarized above can effectively induce apoptosis of bone tumor cells. However, it should be noted that for deep bone tumors, the insufficient penetration of NIR light may lead to treatment failure. In this condition, ultrasound is an effective alternative. In contrast to PDT, sonodynamic therapy is initiated by ultrasound and has a tissue penetration depth of more than 10 cm, which is widely used to ablate deep tumors. In addition to tumor ablation, ultrasound stimulates chondrocyte proliferation, accelerating the maturation of new bone^184^,^185^. Ultrasound-responsive hydrogels doped with HAP particles have been developed for bone regeneration. When triggered by ultrasound, this hydrogel can deliver therapeutic agents on demand. HAP particles as a solid phase to achieve a localized increase in ultrasound responsiveness^186^. Furthermore, silicon nanoparticles have been reported as sound sensitizers for ultrasound-assisted cancer therapy^187^. Based on these findings, we speculate that BG may be ultrasound-responsive, which is a promising treatment method for deep bone tumors.

#### Skin tumor

After surgical resection of a skin tumor, it is usually faced with the risk of large area defects difficult to heal, tissue infection, tumor recurrence, and so on^188^-^190^. PTT is the primary treatment method due to the superficial location of the skin wound. Light-responsive SBGC can inhibit the recurrence of skin tumors and promote the regeneration of damaged tissues.

**Figure 7:**
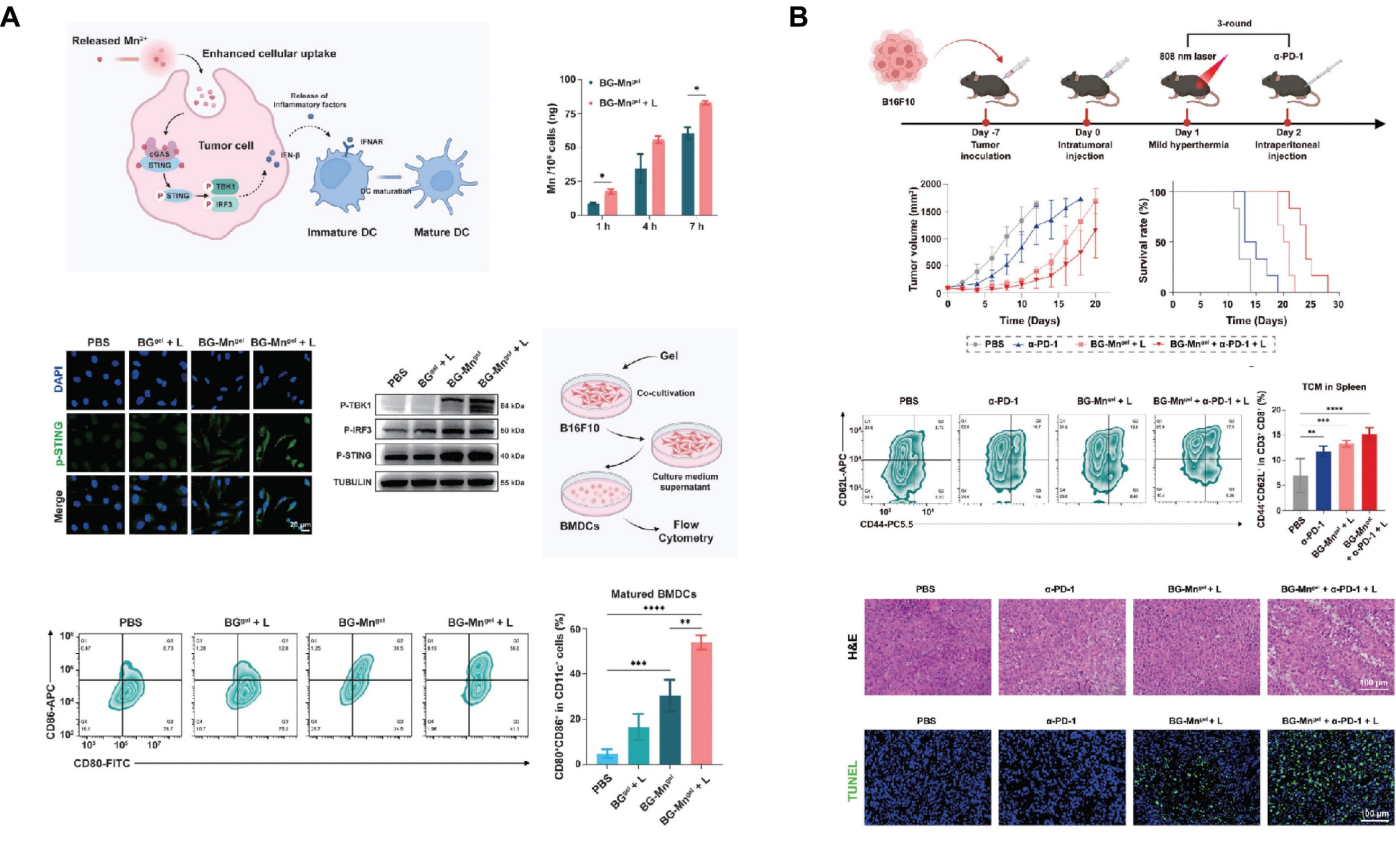
** SBGC in skin tumors:** (A) BG-Mn^gel^ potentiates cGAS-STING activation enhanced by mild hyperthermia *in vitro*. Adapted with permission from 193, copyright 2024 Wiley-VCH. (B) Combination therapy of α-PD-L1 and BG-Mn^gel^ treatment activate long-term immune effects. Adapted with permission from^193^, copyright 2024 Wiley-VCH.

Firstly, MBG can be used as a drug carrier for local administration to prevent tumor recurrence. Based on coaxial electrostatic spinning technology, a bifunctional topical wound dressing was prepared with MBG nanoparticles containing fluorouracil embedded in its core, which not only inhibits cancer but also promotes cell migration and proliferation, thus improving tissue regeneration^191^. Injectable hydrogel-based SBGC is an effective means to treat skin tumors and promote skin wound healing which forms a protective barrier over skin wounds, reducing the risk of infection^192^. Injectable hydrogels (BG-Mn^gel^) formed by crosslinking sodium alginate with Mn-doped BG are used for anti-tumor immunotherapy of melanoma and postoperative wound healing. Mn^2+^ endows BG with excellent light responsiveness. NIR-mediated mild thermotherapy accelerates the release of Mn^2+^ from BG-Mn^gel^ and enhances the uptake of nanoparticles by cancer cells. Further, Mn^2+^ in synergy with mild hyperthermia activates the cGAS-STING immune pathway for tumor immunotherapy (Fig. 7A). The combination of BG-Mn^gel^ and immune checkpoint blockade such as α-PD-L1 can induce a robust T cell memory effect, thereby activating a long-term anti-tumor immune response (Fig. 7B). In addition, BG-Mn^gel^ upregulates the expression of genes related to angiogenesis and promotes skin tissue regeneration in the treatment of full-thickness wounds^193^. Similar hydrogel-based SBGC also achieves reproducible photothermal effects through doping with Ag_2_S nanodots and PDA. While efficiently ablating tumors, the SBGC effectively promotes epithelial reconstruction, collagen deposition, and angiogenesis in normal wounds^194^,^195^.

During PTT, one of the key issues is to ensure that the temperature is determined in situ to completely remove the tumor without damaging the surrounding normal tissues^196^. For this purpose, Nd-Ca-Si bioactive glass system composite hydrogels with tumor ablation and in situ temperature measurement functions were developed. This hydrogel-based SBGC has the best photothermal properties and fluoresces under 808 nm laser irradiation. This provides the optimal PTT temperature for effective tumor treatment with minimal normal tissue damage^148^.

### Breast cancer

As one of the most common malignancies in women, breast cancer is highly invasive and recurrent^197^. SBGC responds to the tumor microenvironment and external stimuli to improve targeting and reduce side effects in breast cancer therapy. For both in situ and metastatic breast cancer, SBGC has shown great potential.

**Figure 8:**
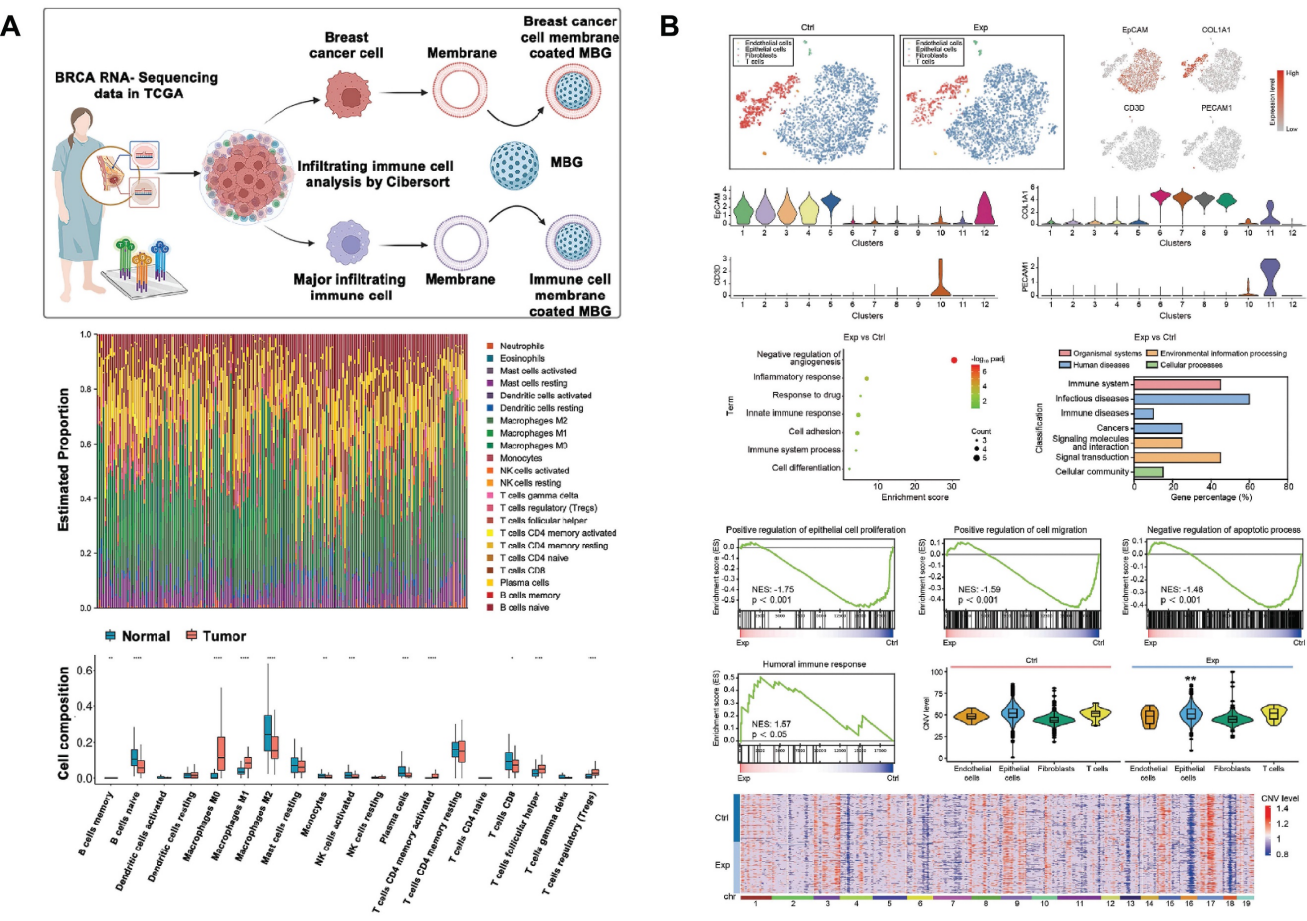
** Application of SBGC in breast cancer:** (A)The cell membrane donor was identified as M0 phenotype macrophage by TCGA analysis. Adapted with permission from 89, copyright 2023 Elsevier. (B) Mechanism of BG@NbSiR-scaffold-based PTT plus anti-PD-L1 immunotherapy depicted by single-cell transcriptomic analysis. Adapted with permission from 204, copyright 2020 Wiley-VCH.

Cell membrane camouflage is an effective targeting method in the treatment of breast cancer lesions in situ. The key to cell membrane camouflage is its ability to mimic natural cellular characteristics, which allows BG to effectively evade recognition and attack by the immune system before reaching the tumor site^198^-^200^. Through The Cancer Genome Atlas Program TCGA database analysis, macrophages of the M0 phenotype have a specific high infiltration fraction in breast cancer patients and are therefore selected as cell-membrane-derived donors (Fig. 8A). Macrophage membrane camouflaged MBG avoids glucose and oxygen, which are found everywhere in the body, and delivers glucose oxidase (Gox) precisely to tumor lesions. In response to glucose and oxygen in the tumor environment, GOx produces ROS to induce oxidative stress and kill tumor cells^89^. Similar studies have utilized red blood cell membrane camouflage to avoid immune clearance, synergizing with chemotherapy, PDT, and PTT for anti-breast cancer therapy^201^.

In addition to treating in situ lesions, another major challenge in breast cancer is the prevention and treatment of cancer metastasis^202^. Bone is the most common site of breast cancer metastasis, resulting in an imbalance between bone resorption and bone formation^203^. This requires the design of multifunctional SBGC that not only simultaneously eliminates primary/metastatic tumors, but also promotes bone tissue regeneration. The immunostimulant R837 and niobium carbide were combined in the BG scaffold (BG@NbSiR). SBGC responds to light stimulation to produce a thermal effect while incorporating PD-L1 immune checkpoint blockade to activate the immune system to eliminate primary and metastatic tumors. In particular, the combination therapy stimulates the host to build a strong long-term immune memory, providing long-term protection against breast cancer, including bone metastases. The degradation products of the scaffold also facilitated the subsequent process of bone regeneration. In further studies, single-cell transcriptomics demonstrated that the combination therapy downregulated the level of copy number variation in tumors and suppressed tumor stem cells, thereby reducing the cancer burden (Fig. 8B)^204^.

The molecular mechanism of SBGC killing tumors is diverse and complex. Firstly, SBGC enhances the level of oxidative stress locally in tumors, inducing the accumulation of ROS and leading to apoptosis or necrosis of tumor cells. The tumor site is a chemical microenvironment that is slightly acidic and overexpresses H_2_O_2_ and glutathione^205^. Ions doped in SBGC respond to the acidic microenvironment of high levels of H_2_O_2_, generating hydroxyl radicals (·OH) with strong oxidizing properties that induce apoptosis in tumor cells^206^. Ions such as Fe, Cu, and Mn can all undergo this reaction in the bone tumor microenvironment^207^,^208^. Among them, Self-expanding cuproptosis further activates immunogenic cell death, triggering a powerful immune response that combines with immune checkpoint blockade to effectively eradicate metastatic tumors^209^. In addition, the SBGC responds to an exogenous magnetic field by releasing excess Ca^2+^, leading to calcium overload and tumor cell death^112^. SBGC can also enhance anti-tumor immune responses and induce long-term T-cell memory by activating the cGAS-STING pathway and PD-L1 immune checkpoint blocking^193^,^204^.

In terms of promoting bone tissue repair, SBGC is mainly adopted to enhance the early osteogenic differentiation of bone mesenchymal stem cells (BMSCs) in bone tumor defects through the activation of osteogenic markers ALP, runt-related transcription factor 2 (Runx2), osteopontin, osteocalcin and bone salivary protein^172^. In addition, the Ca^2+^ released by SBGC activates the calcium-sensitive receptor CaSR of osteoblasts, further promoting bone formation^103^.

In addition to the acidic environment mentioned above, hypoxia is an important tumor microenvironment^210^. Inadequate oxygen supply within the tumor due to rapid growth and irregular vascular network, resulting in local hypoxia^211^. However, SBGC in response to a hypoxic microenvironment has not been reported to treat tumors. Nanoparticles containing the hypoxia-responsive electron acceptor nitroimidazole or hypoxia-sensitive moieties are effective in achieving a response to the hypoxic environment of tumors^212^-^214^. Functionalized MBG can graft organic functional molecules or bioactive substances onto its surface. Therefore, this approach can be used to construct hypoxia-responsive SBGC for targeted tumor therapy. Moreover, inhibition of Na/K-ATPase is a promising cancer therapy. Ultra-small vanadate prodrug nanoparticles modified with bovine serum albumin were synthesized by a combined reductive coordination strategy with natural polyphenol tannic acid. During NIR irradiation, the interaction between vanadate(V) and Na/K-ATPase is selectively enhanced to achieve stronger inhibition of Na/ K-ATPase, resulting in a powerful cell-killing effect^215^.

### Application of SBGC in infectious diseases

Infection control is particularly important in the field of tissue engineering. Although traditional treatments, such as systemic antibiotics, surgical debridement, wound drainage, and implant removal, have been widely used, the effect is not satisfactory^216^-^218^. By adjusting its physical or chemical structure in response to the internal and external environment, SBGC achieves adaptive antibacterial activity. In the treatment of oral infectious diseases, bone tissue infections, and skin wound infections, SBGC demonstrates excellent intelligent antimicrobial and restorative effects.

#### Oral infectious diseases

The oral cavity is a complex and complete micro-ecosystem with specific temperature, humidity, pH, and a rich microbiota, which together maintain the stability of the oral ecosystem^219^-^222^. SBGC responds to environmental changes in the oral cavity for better curative effects. The treatment of oral infections includes caries and periodontitis.

**Figure 9:**
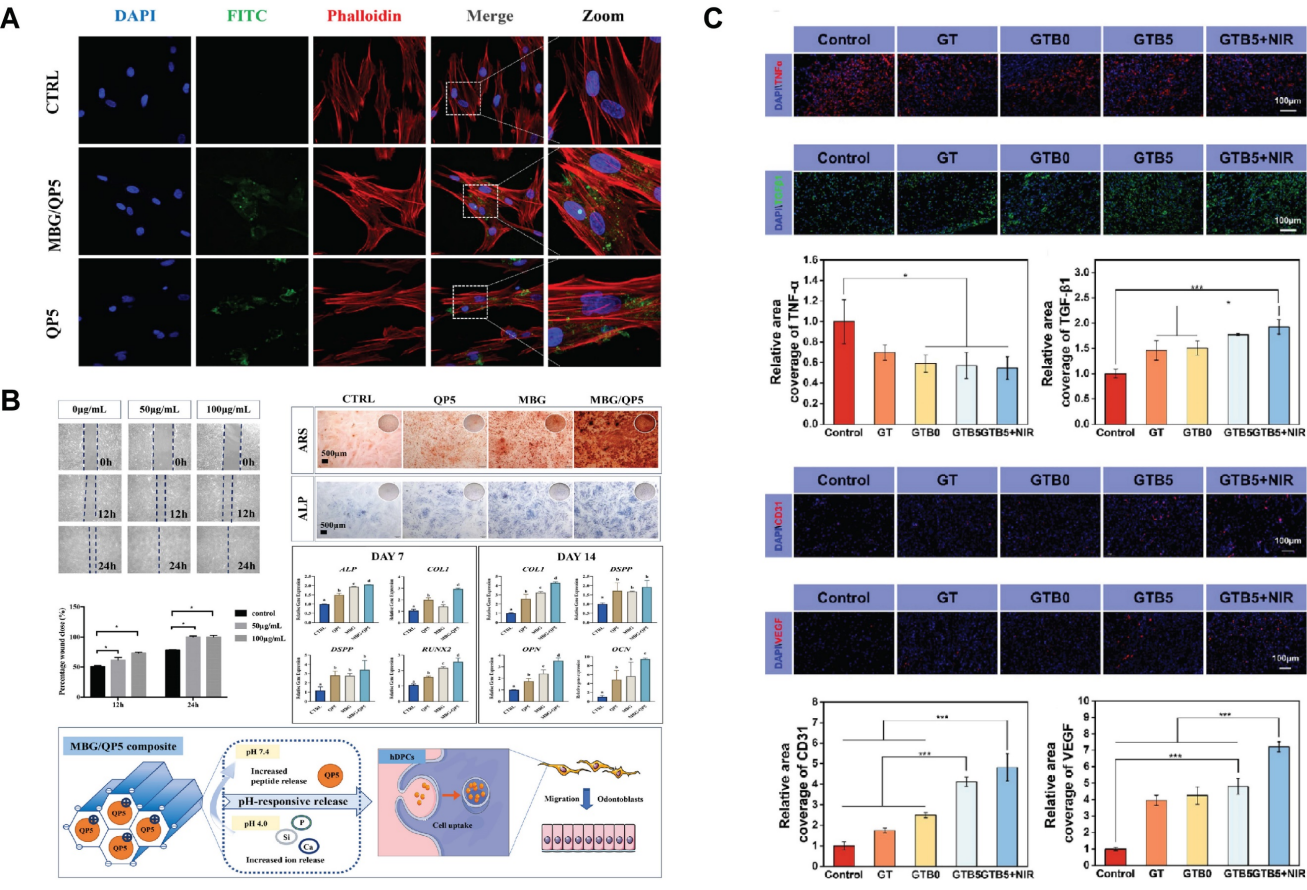
** The application of SBGC in infectious diseases:** (A) MBG/QP5 is internalized and exists in the cytoplasm of human dental pulp cells. Adapted with permission from 225, copyright 2022 SageJournals. (B) The combination of mesoporous bioactive glass MBG/QP5 treated the odontogenic differentiation and mineralization of progenitor pulp cells. Adapted with permission from 225, copyright 2022 SageJournals. (C) Levels of inflammation and angiogenesis in healed skin tissue. Adapted with permission from 242, copyright 2023 ACS publications.

By responding to the acidic environment at the caries site and the constant temperature in the mouth, SBGC treats and restores caries. Plaque formation significantly lowers the local pH level. This acidic environment leads to demineralization of the teeth, which in turn leads to the formation of dental caries. This pathologic process can be used as a design idea for SBGC^223^,^224^. For example, amelogenin-derived peptide QP5 is loaded into the porous structure of MBG by enhanced electrostatic adsorption. MBG/QP5 shows pH responsiveness in acidic environments. Therapeutic ions and functional peptides are released in a sequential cascade and eventually, pH is regulated to a neutral state, preventing further progression of caries. At the same time, MBG/QP5 was internalized by dental pulp cells, which improved the retention of intracellular therapeutic ions and peptides (Fig. 9A). The migration, differentiation, and mineralization of dental pulp cells were further promoted, including the increase of ALP activity, the formation of mineralized nodules and the up-regulation of mineralization-related genes (Fig. 9B)^225^. In addition to pH changes, SBGC also responds to oral temperature to achieve restoration of caries. The temperature-sensitive hydrogel with fluorine-doped bioactive glass (F-BG) as the main body is gelled at room temperature while curing in response to oral temperatures. The cured F-BG has sufficient bond strength and long-term release of fluorine, which effectively prevents microleakage and secondary caries^226^. However, the oral environment is characterized by wetness. When fluids such as blood and saliva are present at the injection site, the coagulation properties of the hydrogel will be affected. Therefore, the ultrasonic scalar is introduced based on temperature response. Sufficient thermal energy is generated by sound waves to rapidly cure the injectable SBGC in situ. This can effectively repair dental tissue defects and promote dentin regeneration^227^.

Periodontitis is a chronic inflammatory disease that invades the gums and periodontal tissues, which is the leading cause of tooth loss in adults. Guided tissue regeneration (GTR) and guided bone regeneration (GBR) are effective methods to promote periodontal tissue regeneration using physical barriers^228^,^229^. Gel-based SBGC gelatinizes in response to light stimulation, thus providing the barrier function required for GBR membranes. A light-responsive SBGC gel (MBGN-MNC1/MHA) loaded with minocycline hydrochloride can be injected directly into the irregular periodontal defect area, which then responds to ultraviolet light by rapidly solidifying and molding. MBGN-MNC1/MHA significantly decreased the expression of macrophage inflammatory factors IL-6 and TNF-α, while promoting the expression of osteogenesis-related genes including ALP, Runx2, and OPN^230^. There is no doubt that SBGC shows potential in the treatment of irregular periodontal defects caused by periodontitis. However, due to its easy degradation and poor mechanical properties, the barrier function of hydrogels needs further investigation compared with traditional GBR membranes.

#### Bone infection

Bone tissue infections are common after fracture, bone grafting, or bone reconstruction surgery. Any surgical intervention involving the implantation of biomaterials and the application of fixation devices carries the risk of implant-associated infections^231^-^233^. Antimicrobial drug delivery and photothermal sterilization are the main modalities used by SBGC to treat bone tissue infections.

The microenvironment of infected bone tissue is slightly acidic due to bacterial metabolism^234^. SBGC responds to this microenvironment to ensure that antibiotics are released at the site of infection. This improves the bioavailability of the drug and avoids the side effects associated with systemic antibiotic use^235^. For example, BG-HA@CS has pH-responsive drug-release properties. The expansion of chitosan in an acidic environment opens holes in the hydroxyapatite shell, accelerating the release of vancomycin. With the release of the drug, the unconverted BG dissolves, raising the local pH. Changes in pH, in turn, regulate the solubilization behavior of chitosan, enabling self-regulated drug release^236^. Vancomycin-loaded zeolitic imidazolate frameworks-8 (ZIF-8@VAN) are deposited on a 3D-printed BG scaffold in another study. ZIF is acid-responsive and degrades at the site of infection to release antibiotics. Also, Zn^2+^ released by ZIF has antimicrobial activity^237^. Although the osteogenic effect of BG has been recognized, the above studies do not investigate the osteogenic effect after anti-infection.

In addition to antibiotics, metal ions also show excellent antibacterial effects^238^. Moreover, some metal ions also have excellent light responsiveness and can destroy bacteria through the photothermal effect^239^. Ce-BG showed excellent photothermal bactericidal properties under 808 nm laser irradiation while promoting osteoblast proliferation^240^. Combining the antimicrobial effect of metal ions with photothermal sterilization is expected to realize efficient antimicrobial treatment without antibiotics.

#### Skin wound infection

Infection control is an important step to ensure effective wound repair. Infection causes inflammation and tissue damage, which can delay healing time^241^. Light-responsive hydrogel-based SBGC has broad application prospects in treating wound infection and promoting wound healing.

Hydrogel provides the right microenvironment for repairing skin damage. For example, in the composite hydrogel containing Fe-BGs, the chelation of Fe^3+^ with tannin endowed the hydrogel with good photothermal antimicrobial ability, and the response to NIR effectively activated the angiogenic ability (Fig. 9C)^242^. In another study, Ag_2_S nano-dots conjugated with iron-doped bioactive glass nanoparticles (BGN-Fe-Ag_2_S) were incorporated into polyethylene glycol diacrylate (PEGDA) in situ gel to develop a photoactivated injectable hydrogel. In a full-thickness skin wound model, this hydrogel can not only eliminate multidrug-resistant bacteria by hydrolysis of the bioactive glass but also accelerate wound healing and regenerate more skin attachments^194^. The Cu ion has effective antibacterial activity and destroys the cell membrane through electrostatic interaction between its positive charge and the negatively charged microbial cell membrane^243^. Cu-coordination PDA wrapped into bioactive BGN@PDA-Cu nanoparticles can significantly reverse the inflammatory phenotype of macrophages and promote angiogenesis by activating hypoxia-inducing factor HIF-1α and heat shock protein HSP90 pathway^244^. In addition, due to its photothermal properties and nitric oxide release activity, PDA is often used in skin injury repair. NO, Ca, and Si ions released by PDA-BGNPs modified with β-cyclodextrin have the benefit of regulating inflammation, promoting fibroblast proliferation, and stimulating angiogenesis^245^.

#### Other infectious diseases

In addition to localized tissue infections, SBGC also shows potential applications for the surveillance of systemic infectious diseases. Specific serum biomarkers are critical for early diagnosis of disease and assessment of disease progression. For example, procalcitonin (PCT), as a biomarker of sepsis, is beneficial for early diagnosis in critically ill patients. Researchers have proposed a sandwich-type electrochemical immunosensor that utilizes Pd nanoparticle-loaded Fe_3_S_4_ as a signal indicator and functionalized pineal MBG as a marker for signal amplification. Based on the response of the signal-off mode with sensitive changes in the current signal, this SBGC sensor can detect PCT with high sensitivity. This technique is suitable for the sensitive detection of PCT and the clinical diagnosis of other disease biomarkers and cells^246^.

The antibacterial mechanism of SBGC is firstly to induce oxidative stress by releasing ions to generate reactive oxygen species, which leads to DNA, protein, and lipid damage of pathogens^247^,^248^. In addition to reducing the expression of macrophage inflammatory factors IL-6 and TNF-α^230^, SBGC can also directly regulate the transformation of macrophages from m1 type to m2 type by promoting autophagy and weakening the inhibition of autophagy flow. In addition, SBGC also indirectly regulates the polarization phenotype of macrophages by reducing the activation of NF-κb in bone marrow mesenchymal stem cells and restoring their immunomodulatory capacity^249^. In promoting soft tissue regeneration, SBGC activates the eNOS/NO signaling pathway to enhance the adhesion, migration, and differentiation of endothelial cells and promotes angiogenesis^102^, as well as the proliferation of fibroblasts, which promotes epithelial reconstruction and collagen deposition in normal wounds.

### Application of SBGC in diabetes and its complications

Diabetes is a common metabolic disorder characterized by persistent hyperglycemia. The diabetes microenvironment is complex and variable, typically characterized by chronic inflammation, high oxidative stress, and local blood flow disturbances that lead to tissue dysfunction and prevent normal wound healing^250^-^252^. By responding to the diabetic microenvironment, SBGC delivers insulin to help regulate systemic blood glucose levels. At the same time, SBGC also accelerates the healing of difficult-to-heal wounds and promotes tissue regeneration and repair during localized treatment.

#### Insulin delivery

**Figure 10:**
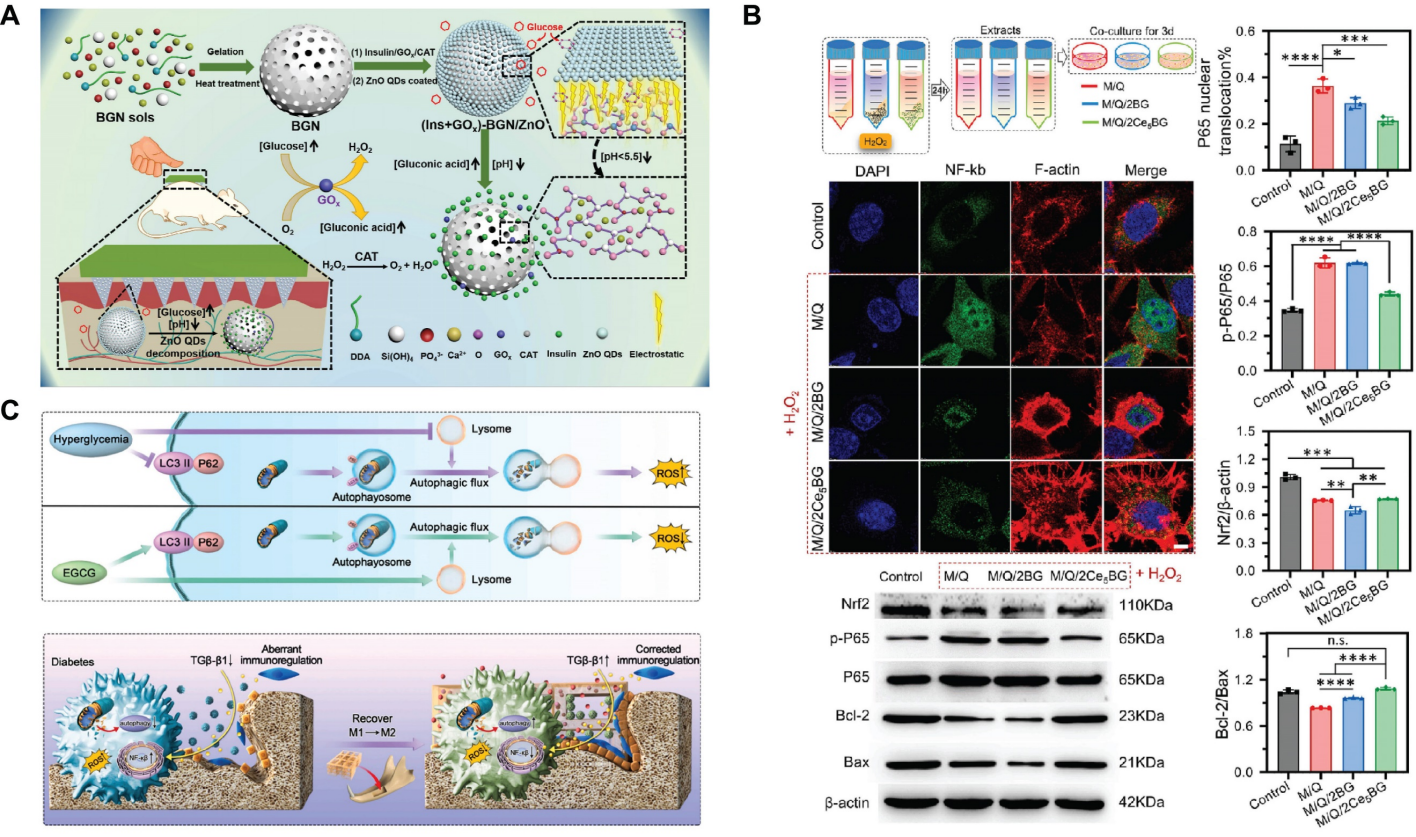
** Application of SBGC in diabetes and its complications:** (A) Schematic preparation of glucose-mediated microneedles integrated with ZnO QDs capped MBG for transdermal delivery of insulin. Adapted with permission from 253, copyright 2018 ACS publications. (B) M/Q/CeBG hydrogel attenuates H_2_O_2_-induced L929 cell injury and apoptosis. Adapted with permission from 66, copyright 2024 Elsevier. (C) Schematic illustration of ABBSG@EGCG fabrication by functional Borosilicate bioactive glass and epigallocatechin gallate for healing of alveolar bone defects in diabetes. Adapted with permission from 249, copyright 2023 Elsevier.

Daily subcutaneous insulin injections are the standard diabetes treatment, which is cumbersome and invasive. Therefore, a smart delivery system that is minimally invasive, painless, and controls the release of insulin with blood sugar levels is essential. A pH-responsive and glucose-mediated SBGC delivery system is developed to enable controlled and painless insulin administration. Insulin and glucose response factors (GOx/CAT) are encapsulated in MBG, and ZnO QDs act as pH response switches to coat MBG nanopores. GOx/CAT catalyzes the production of gluconic acid from glucose, resulting in a local decrease in pH. The ZnO QDs dissolve under acidic conditions, triggering the disassembly of pH-sensitive MBG, which releases pre-loaded insulin (Fig. 10A)^253^. Similar studies control insulin release with a complex enzyme layer of polyethyleneimine, GOx, and CAT. The complex enzyme layer converts glucose to gluconic acid. Changes in pH in the microenvironment lead to structural disruption of the complex enzyme layer, realizing the gradual release of insulin^254^.

#### Diabetic ulcer

Diabetic ulcers heal more slowly and are more susceptible to infection than normal wounds. Oxidative stress caused by excess ROS is the key factor in delayed healing. Unbalanced ROS not only delays angiogenesis but also leads to a long-term state of excessive inflammation that impedes wound healing. In addition, persistent high blood sugar in diabetic wounds makes them more susceptible to microbial infections^250^,^255^,^256^.

First, SBGC responds to high levels of ROS in the lesion environment, reducing oxidative stress, reversing inflammation, and promoting healing of difficult-to-heal wounds. For example, CeBG-based hydrogel responds to and scavenges ROS via redox reactions and attenuates H_2_O_2_-induced cell damage and apoptosis (Fig. 10B). At the same time, Ce-BG hydrogel stimulates the proliferation and migration of dermal fibroblasts^66^. Similarly, Co-doped BG also responds to ROS. Co_3_O_4_ microcrystals are formed in situ by the chemical reaction of cobalt with ROS (mainly H_2_O_2_), which plays a decisive role in clearing various ROS and inhibiting the inflammatory cascade. Moreover, cobalt shows specific therapeutic properties that promote angiogenesis, increasing endothelial migration and tubule formation^247^.

In addition to overproducing ROS, high glucose levels exacerbate wound bacterial infections. The high-glucose environment provides rich nutrients for the bacteria and suppresses the immune system's response, leading to more severe and stubborn infections^257^-^259^. Thus, a non-antibiotic drug formulation is proposed for diabetic wound infection. In this scheme, gallic acid (GA) and BG produce synergistic effects through multiple biological pathways. On the one hand, GA has an antibacterial effect and further enhances antibacterial ability through a photothermal effect. On the other hand, Si released by BG simultaneously regulates inflammation and promotes angiogenesis^260^. Although the antioxidant capacity of gallic acid has been extensively documented, the effect of GA@BG in controlling oxidative stress on diabetic-infected wounds is not described in detail. In another study, MnO_2_ is deposited on the surface of MBG to construct SBGC (MnO_2_@PDA-BGs) with dual antioxidant and antibacterial functions. MnO_2_@PDA-BGs show a photothermal effect under acidic conditions, showing excellent antibacterial properties. Meanwhile, MnO_2_ reduces oxidative stress by scavenging free radicals, releasing oxygen to improve the hypoxic microenvironment of the wound, which helps promote vascularization and collagen deposition^261^.

#### Diabetes with periodontitis

Periodontitis is the sixth most common complication of diabetes^262^. Plaque is the initiator of periodontitis, and diabetes exacerbates the localized inflammation caused by plaque. What's more, the high glucose environment and the formation of advanced glycosylation end products (AGEs) induce ROS accumulation, further exacerbating the inflammatory response. In addition, the high glucose environment inhibited the bone regeneration capacity of BMSCs, leading to alveolar bone resorption and tooth loosening^263^-^266^. Therefore, SBGC needs to respond adaptively to this specific microenvironment to slow or reverse the process of periodontitis.

For hyperglycemia and oxidative stress in diabetes, a glucose-H_2_O_2_-responsive SBGC scaffold is prepared. The scaffold releases epigallocatechin gallate (EGCG) in the diabetic environment, synergically regulating abnormal inflammation by directly regulating the polarization state of macrophages. Further studies reveal that EGCG directly regulates the macrophage transition from M1-type to M2-type by promoting autophagy and attenuating the inhibition of autophagic flow. In addition, EGCG also indirectly regulates the polarization phenotype of macrophages by reducing the activation of NF-κb in BMSCs and restoring their immunomodulatory ability (Fig. 10C). Altered macrophage phenotype reduces local inflammation and thus improves the ability to repair diabetic alveolar bone^249^.

### Applications of SBGC in other diseases

#### Acute bleeding

Acute bleeding refers to a condition in which a large amount of blood is lost in a short period, usually caused by trauma, surgery, or disease. Control of bleeding is a critical step in the management of acute trauma. Therefore, the development of rapid and efficient hemostatic methods is particularly important^267^-^269^.

Shape memory SBGC offers the possibility of rapid and effective hemostasis. A bioactive self-expanding cold gel (BGNCs) composed of highly flexible bioactive glass nanofibers and citric acid crosslinked polyvinyl alcohol. Due to the flexibility of BG nanofibers, BGNCs exhibit rapid self-expansion, injectability, and superelasticity, and firmly adhere to various tissues. These excellent properties make BGNCs exhibit excellent hemostatic performance in liver incompressible hemorrhage models and arterial injury models^270^. A similar shape-memory cryogel cross-links QCS to BG, which is capable of rapidly absorbing blood and regaining its shape upon compression, forming a physical barrier to block bleeding sites^155^.

#### Inflammatory bowel disease

The key to the treatment of inflammatory bowel disease is to relieve inflammation and promote intestinal tissue regeneration. Core-shell microspheres of zein/sodium alginate are used for oral delivery of BG. The microspheres dissolve in a simulated intestinal environment, which helps prevent BG from dissolving prematurely in the stomach. In response to specific enzymes in the intestinal environment, the zein and alginate in the microspheres are degraded by the enzymes, releasing BG to exert therapeutic effects. In mouse models of acute and chronic colitis, BG significantly reduced intestinal inflammation, promoted epithelial tissue regeneration, and partially restored microbiota homeostasis. Thus, the targeted release of BG in the intestine provides a novel, cost-effective therapeutic approach for the effective treatment of inflammatory bowel disease^271^.

## Perspectives

Although SBGC has shown great potential in tissue engineering and medical therapeutics, several challenges remain in the field. First, the microenvironment changes dynamically during disease progression, including pH, oxygen levels, levels of inflammatory factors and metabolites. Therefore, the design of SBGC needs to take these dynamic changes into account to ensure that they can still perform their functions effectively under different pathologic conditions. Second, the biocompatibility and immune response of SBGC *in vivo* is also a key issue. Although current studies have demonstrated the efficacy of SBGC in some applications, the immune response and the effect of biodegradation products on the organism after long-term implantation still need to be studied. Finally, the smart response capability of SBGC relies on their interaction with cells and tissues. To improve its performance, a deeper understanding of the mechanisms of cell-material interactions is needed.

Individualized treatment is becoming a trend. Customization of the SBGC with in-depth study of the patient's genetic information, cellular status, and lesion characteristics to enable a more precise response to the disease state. Combining sensor technology and biofeedback systems, the SBGC monitors changes in the microenvironment in real time, such as changes in specific disease signals and inflammation levels. Based on this information, the SBGC provides feedback and adjusts treatment steps to maximize the effectiveness of treatment. In addition, appropriate signaling indicators and markers are selected for continuous and unencumbered remote monitoring of disease states and biomarkers. Examples include monitoring blood glucose levels for early diagnosis and subsequent treatment of diabetes, detecting troponin to help predict the risk of myocardial infarction and other cardiovascular events, and detecting tumor markers in serum for early screening and diagnosis of cancer, among others^272^-^275^.

3D-printed technology has greatly advanced the field of tissue engineering, brought great potential, and promised to the field of personalized medicine and injury repair. However, traditional 3D-printed still has some limitations, such as a lack of dynamism and responsiveness^276^. To overcome these limitations, researchers at the Massachusetts Institute of Technology (MIT) came up with the concept of 4D-printed technology in 2013. Unlike traditional 3D-printed, 4D-printed considers changes in the material over time. The printed material automatically deforms or changes its properties under certain conditions^277^-^279^. The technology of BG in 3D-printed is relatively mature, but research in 4D-printed has not been reported. 4D-printed bioactive glass responds dynamically to the environment or stimuli to accommodate a variety of irregular defective morphologies. While 4D-printed adds design complexity, fabricated stimulus-responsive structures provide dynamic, reprogrammable deformations or actions that mimic the complex physical, biochemical, and mechanical processes of tissues^280^,^281^.

In addition to new preparation methods, novel types of materials have been developed recently including programmable, bioelectronic, and cell membrane coating materials. Combining SBGC with new materials will gain more functions which will better repair complex tissue defects. For example, bioelectronic materials have the problem of no detectable signal or low signal sensitivity; while SBGC has the function of generating acceptable signals such as temperature and pH by responding microenvironment, improving the sensitivity of bioelectronic materials. Meanwhile, the physical and chemical signals generated by smart response will also participate in the construction of programmable biomaterials, achieving dynamic construction of materials *in vivo*. In addition, to address the problem of low targeting of SBGC for tumor treatment, cell membranes can be wrapped around the SBGC particles to construct cell membrane-encapsulated materials to achieve precise targeting.

Most importantly, although this paper summarizes the SBGC, it is worth noting that most of the intelligent response to stimuli is the role played by other components of the composite. Therefore, in the future, the construction of real smart materials, in which only BG is involved, is the research direction.

## Figures and Tables

**Table 1:** Internal microenvironment responsive SBGC

Response type of the SBGC	Functional component	How to respond	Ref.
pH-responsive	ZnO QDs	ZnO QDs are released rapidly at pH 4.0.	38
	HAP	MBG spontaneously mineralizes to form HAP, rapidly degrading at pH = 4.0.	39
	DOX	DOX desorption from MBG at pH = 4.3. Ca^2+^ chelated DOX is released as Ca^2+^ dissolves from MBG at pH = 4.0.	41 42
	PLGA	At pH ~ 5.5, the PLGA covalent bond is broken.	48
	AIEgens	The emission wavelength changes reversibly with pH from 2.0 to 4.0.	51,52
ROS-responsive SBGC	Gallic acid PTA	Reactive hydroxyl groups of polyphenols donate hydrogen atoms to free radicals to neutralize free radical activity	62,63 65
	Ce	Redox reaction Redox reaction	66-69
	Mo		71
Temperature-responsive SBGC	PIB CS	It changes from liquid to gel at body temperature. It changes from liquid to gel at body temperature.	76 78-80
Enzyme-responsive SBGC	ATP ε-PL	The increased ACP causes hydrolysis of the phosphate bond of ATP. ε-PL hydrolysis in the presence of protease.	84 85

**Table 2:** Exogenous stimulus-responsive SBGC

Response type of the SBGC	Functional component	How to respond	Ref.
Electrical stimulus-responsive SBGC	BG	Polarization changes the charge arrangement inside BG	96-98
	CNT	CNT forms a conductive network in BG	99,100
	KNN	Piezoelectric effect	102,158,159
	PVFT	Piezoelectric effect	103
	BaTiO_3_	Piezoelectric effect	104
Magnetic field-responsive SBGC	Fe_3_O_4_	Magnetocaloric effect	108,110,112,116
	MnO_2_, Fe_2_O_3_	Magnetocaloric effect	109
	γ-Fe_2_O_3_	Magnetocaloric effect	111
	α-Fe_2_O_3_	Magnetocaloric effect	115
	BF	Magnetocaloric effect	117
Light-responsive SBGC	Fe、Mn、Cu、Mo	Photothermal effect caused by SPR	130
	Bi	Photothermal effect caused by SPR	127,131
	Cu	Photothermal effect caused by SPR	132
	Nb2C	Photothermal effect in response to NIR	135
	carbon dots	Photothermal effect in response to NIR	136
	CuFeSe_2_	Photothermal effect in response to NIR	137
	DTC	Photothermal effect in response to NIR	140
	Hematin	Photothermal effect in response to NIR	141
	PDA	Photothermal effect in response to NIR	118-120]
	coumarin	UV light (>310 nm) induced photodimerization of coumarin, while UV light (250nm) induced lysis of coumarin dimer and regenerated coumarin monomer	139
Shape memory SBGC	PCL-TES	The triethoxy-silyl group is hydrolyzed and condensed with the silicon hydroxyl group of BG.	151
	chitosan	Shape memory properties are triggered by hydration.	152-155
	Gelatin-hyaluronic acid hydrogel	Shape memory properties are triggered by hydration	156

**Table 3:** The applications of SBGC

Type of Disease	Functional component	treatment strategy	Ref.
Bone tumor	DOX	Drug delivery	42,165,166
	UCNPs	Bioimaging	167,170-173
	Cu, Mn	CDT	176
	SrAl_2_O_4_: Eu, Dy	PDT	177
	Cu	PTT	180
	Mn	PTT	181
	Fe_3_O_4_	MTT	112
Skin tumor	Mn	PTT, immunotherapy	193
	Ag_2_S nanodots	PTT	194
	PDA	PTT	195
	Nd	PTT, temperature monitoring	148
Breast cancer	Gox	CDT	89
	Cu, PDA	PDT, PTT	201
	R837, Nb2C	PTT, immunotherapy	204
Oral infectious diseases	QP5	Drug delivery	225
	pluronic F127	Thermal response	226,227
	hyaluronic acid	Light response	230
Bone infection	chitosan	pH-response drug delivery	236
	ZIF	pH-response drug delivery	237
	Ce	Photothermal antibacterial	240
Skin wound infection	Fe, tannin	Photothermal antibacterial	242
	PDA	Photothermal antibacterial	245
Diabetes	ZnO QDs, GOx/CAT	Insulin delivery	253
	GOx, CAT	Insulin delivery	254
Diabetic ulcer	Ce	ROS is cleared by redox reaction	66
	Co	ROS is cleared by redox reaction	247
	GA	Photothermal antibacterial	260
	MnO_2_, PDA	Photothermal antibacterial	261
Diabetes with periodontitis	EGCG	ROS is cleared by redox reaction	249
Acute bleeding	polyvinyl alcohol	Shape memory	270
	QCS	Shape memory	155
Inflammatory bowel disease	zein/sodium alginate	Enzyme-response to drug delivery	271

## Abbreviations

AIEgens: aggregation-induced emission luminogens
ATP: adenosine triphosphate
ALP: alkaline phosphatase
ACP: acid phosphatase
AGEs: advanced glycation end products
BG: bioactive glass
Bi: Bismuth
BF: barium ferrite
BMSCs: bone marrow mesenchymal stem cells
Ce: Cerium
CNT: carbon nanotube
CDT: chemodynamic therapy
CAT: catalase
Co: Cobalt
DOX: Doxorubicin
ES: electrical stimulation
EGCG: epigallocatechin gallate
Gox: glucose oxidase
GTR: guided tissue regeneration
GA: gallic acid
GBR: guided bone regeneration
HAP: hydroxyapatite
H_2_O_2_: hydrogen peroxide
KNN: potassium-sodium niobate
LCST: lower critical dissolution temperature
MBG: mesoporous bioactive glass
Mo: Molybdenum
MTT: magnetothermal therapy
NIR: near-infrared
PLGA: poly-L-glutamic acid
PLGA: poly-L-glutamic acid
PTA: polytannic acid
PIB: p (N-isopropylacrylamide-co-butyl methylacrylate)
PDA: polydopamine
PVFT: polyvinylidene difluoride
PDT: photodynamic therapy
PTT: photothermal therapy
PCT: procalcitonin
QCS: quaternized chitosan
ROS: reactive oxygen species
Runx2: runt-related transcription factor 2
SBGC: smart bioactive glass composites
SPR: surface plasmon resonance
UCNPs: upconversion nanoparticles
UV: ultraviolet
ZnO QDs: ZnO quantum dots
ZIF: zeolitic imidazolate frameworks
ε-PL: ε-polylysine

## References

[1] Baino F, Fiorilli S, Vitale-Brovarone C (2016). Bioactive glass-based materials with hierarchical porosity for medical applications: Review of recent advances. Acta Biomater.

[2] Simila HO, Boccaccini AR (2022). Sol-gel bioactive glass containing biomaterials for restorative dentistry: A review. Dent Mater.

[3] Vallet-Regi M, Salinas AJ (2021). Mesoporous bioactive glasses for regenerative medicine. Mater Today Bio.

[4] Turner J, Nandakumar A, Anilbhai N, Boccaccini AR, Jones JR, Jell G (2023). The effect of Si species released from bioactive glasses on cell behaviour: A quantitative review. Acta Biomater.

[5] Shearer A, Montazerian M, Sly JJ, Hill RG, Mauro JC (2023). Trends and perspectives on the commercialization of bioactive glasses. Acta Biomater.

[6] Aslam AA, Akram J, Mehmood RA, Mubarak A, Khatoon A, Akbar U (2023). Boron-based bioactive glasses: Properties, processing, characterization and applications. Ceram Int.

[7] Zhou D, Yan X, Xiao L, Wang J, Wei J (2024). Gold capped mesoporous bioactive glass guides bone regeneration via BMSCs recruitment and drug adaptive release. Chem Eng J.

[8] Gu J, Liu X, Cui P, Yi X (2023). Multifunctional bioactive glasses with spontaneous degradation for simultaneous osteosarcoma therapy and bone regeneration. Biomater Adv.

[9] Zambon A, Malavasi G, Pallini A, Fraulini F, Lusvardi G (2021). Cerium Containing Bioactive Glasses: A Review. ACS Biomater Sci Eng.

[10] Cui Y, Hong S, Jiang W, Li X, Zhou X, He X (2024). Engineering mesoporous bioactive glasses for emerging stimuli-responsive drug delivery and theranostic applications. Bioact Mater.

[11] Zhu H, Monavari M, Zheng K, Distler T, Ouyang L, Heid S (2022). 3D Bioprinting of Multifunctional Dynamic Nanocomposite Bioinks Incorporating Cu-Doped Mesoporous Bioactive Glass Nanoparticles for Bone Tissue Engineering. Small.

[12] Tabia Z, Mabrouk KE, Bricha M, Nouneh K (2019). Mesoporous bioactive glass nanoparticles doped with magnesium: drug delivery and acellular in vitro bioactivity. RSC Adv.

[13] El-Fiqi A, Kim T-H, Kim M, Eltohamy M, Won J-E, Lee E-J (2012). Capacity of mesoporous bioactive glass nanoparticles to deliver therapeutic molecules. Nanoscale.

[14] Anand A, Sengupta S, Kaňková H, Švančárková A, Beltrán AM, Galusek D (2022). Influence of Copper-Strontium Co-Doping on Bioactivity, Cytotoxicity and Antibacterial Activity of Mesoporous Bioactive Glass. Gels.

[15] Zhao S, Zhang J, Zhu M, Zhang Y, Liu Z, Ma Y (2015). Effects of functional groups on the structure, physicochem-ical and biological properties of mesoporous bioactive glass scaffolds. J Mater Chem B.

[16] Verné E, Ferraris S, Vitale-Brovarone C, Spriano S, Bianchi CL, Naldoni A (2010). Alkaline phosphatase grafting on bioactive glasses and glass ceramics. Acta Biomater.

[17] Gupta N, Singh A, Dey N, Chattopadhyay S, Joseph JP, Gupta D (2021). Pathway-Driven Peptide-Bioglass Nanocomposites as the Dynamic and Self-Healable Matrix. Chem Mater.

[18] Jiang S, Zhang Y, Shu Y, Wu Z, Cao W, Huang W (2017). Amino-functionalized mesoporous bioactive glass for drug delivery. Biomed Mater.

[19] Zhu Y, Ma Z, Kong L, He Y, Chan HF, Li H (2020). Modulation of macrophages by bioactive glass/sodium alginate hy-drogel is crucial in skin regeneration enhancement. Biomaterials.

[20] Moreira CDF, Carvalho SM, Mansur HS, Pereira MM (2016). Thermogelling chitosan-collagen-bioactive glass nanoparticle hybrids as potential injectable systems for tissue engineering. Mater Sci Eng C.

[21] Almeida AC, Vale AC, Reis RL, Alves NM (2020). Bioactive and adhesive properties of multilayered coatings based on catechol-functionalized chitosan/hyaluronic acid and bioactive glass nanoparticles. Int J Biol Macromol.

[22] Fu S, Du X, Zhu M, Tian Z, Wei D, Zhu Y (2019). 3D printing of layered mesoporous bioactive glass/sodium alginate-sodium alginate scaffolds with controllable dual-drug release behaviors. Biomed Mater.

[23] Liu J, Zhou X, Zhang Y, Zhu W, Wang A, Xu M (2022). Rapid hemostasis and excellent antibacterial cerium-containing mesoporous bioactive glass/chitosan composite sponge for hemostatic material. Mater Today Chem.

[24] Sun Y, Chen CS, Fu J (2012). Forcing Stem Cells to Behave: A Biophysical Perspective of the Cellular Microenvironment. Annu Rev Biophys.

[25] Sato A, Rahman NIA, Shimizu A, Ogita H (2021). Cell-to-cell contact-mediated regulation of tumor behavior in the tumor microenvironment. Cancer Sci.

[26] Keung AJ, Kumar S, Schaffer DV (2010). Presentation Counts: Microenvironmental Regulation of Stem Cells by Biophysical and Material Cues. Annu Rev Cell Dev Biol.

[27] Barthes J, Özçelik H, Hindié M, Ndreu-Halili A, Hasan A, Vrana NE (2014). Cell microenvironment engineering and monitoring for tissue engineering and regenerative medicine: the recent advances. BioMed Res Int.

[28] Yao Y, Zhang H, Wang Z, Ding J, Wang S, Huang B (2019). Reactive oxygen species (ROS)-responsive biomaterials mediate tissue microenvironments and tissue regeneration. J Mater Chem B.

[29] Kato Y, Ozawa S, Miyamoto C, Maehata Y, Suzuki A, Maeda T (2013). Acidic extracellular microenvironment and cancer. Cancer Cell Int.

[30] Deng Z, Li J, Liu H, Luo T, Yang Y, Yang M (2022). A light-controlled DNA nanothermometer for temperature sensing in the cellular membrane microenvironment. Biosens Bioelectron.

[31] Barmaki S, Jokinen V, Obermaier D, Blokhina D, Korhonen M, Ras RHA (2018). A microfluidic oxygen sink to create a targeted cellular hypoxic microenvironment under ambient atmospheric conditions. Acta Biomater.

[32] Wang Y, Wu Y, Long L, Yang L, Fu D, Hu C (2021). Inflammation-Responsive Drug-Loaded Hydrogels with Sequential Hemostasis, Antibacterial, and Anti-Inflammatory Behavior for Chronically Infected Diabetic Wound Treatment. ACS Appl Mater Interfaces.

[33] Yan L-X, Chen L-J, Zhao X, Yan X-P (2020). pH Switchable Nanoplatform for In Vivo Persistent Luminescence Imaging and Precise Photothermal Therapy of Bacterial Infection. Adv Funct Mater.

[34] Liu W, Wang T, Yang C, Darvell BW, Wu J, Lin K (2016). Alkaline biodegradable implants for osteoporotic bone defects—importance of microenvironment pH. Osteoporos Int.

[35] Gerweck LE, Seetharaman K (1996). Cellular pH gradient in tumor versus normal tissue: potential exploitation for the treatment of cancer. Cancer Res.

[36] Zhang J, Wu D, Li M-F, Feng J (2015). Multifunctional Mesoporous Silica Nanoparticles Based on Charge-Reversal Plug-Gate Nanovalves and Acid-Decomposable ZnO Quantum Dots for Intracellular Drug Delivery. ACS Appl Mater Interfaces.

[37] Wu S, Huang X, Du X (2015). pH- and redox-triggered synergistic controlled release of a ZnO-gated hollow mesoporous silica drug delivery system. J Mater Chem B.

[38] Zheng K, Lu M, Rutkowski B, Dai X, Yang Y, Taccardi N (2016). ZnO quantum dots modified bioactive glass nanoparticles with pH-sensitive release of Zn ions, fluorescence, antibacterial and osteogenic properties. J Mater Chem B.

[39] Yang C, Guo W, Cui L, Xiang D, Cai K, Lin H (2014). pH-responsive controlled-release system based on mesoporous bioglass materials capped with mineralized hydroxyapatite. Mater Sci Eng C.

[40] Li Y, Zhi X, Lin J, You X, Yuan J (2017). Preparation and characterization of DOX loaded keratin nanoparticles for pH/GSH dual responsive release. Mater Sci Eng C.

[41] Wang X, Wang G, Zhang Y (2017). Research on the biological activity and doxorubicin release behavior *in vitro* of mesoporous bioactive SiO2-CaO-P2O5 glass nanospheres. Appl Surf Sci.

[42] Wu C, Fan W, Chang J (2013). Functional mesoporous bioactive glass nanospheres: synthesis, high loading efficiency, controllable delivery of doxorubicin and inhibitory effect on bone cancer cells. J Mater Chem B.

[43] Zeng X, Liu G, Tao W, Ma Y, Zhang X, He F (2017). A Drug-Self-Gated Mesoporous Antitumor Nanoplatform Based on pH-Sensitive Dynamic Covalent Bond. Adv Funct Mater.

[44] Wang M, Xu L, Lin M, Li Z, Sun J (2021). Fabrication of reversible pH-responsive aggregation-induced emission luminogens assisted by a block copolymer via a dynamic covalent bond. Polym Chem.

[45] Tao A, Huang GL, Igarashi K, Hong T, Liao S, Stellacci F (2020). Polymeric Micelles Loading Proteins through Concurrent Ion Complexation and pH-Cleavable Covalent Bonding for In Vivo Delivery. Macromol Biosci.

[46] Bennewitz MF, Lobo TL, Nkansah MK, Ulas G, Brudvig GW, Shapiro EM (2011). Biocompatible and pH-Sensitive PLGA Encapsulated MnO Nanocrystals for Molecular and Cellular MRI. ACS Nano.

[47] Zolnik BS, Burgess DJ (2007). Effect of acidic pH on PLGA microsphere degradation and release. J Controlled Release.

[48] Das MP, Pandey G, Neppolian B, Das J (2021). Design of poly-l-glutamic acid embedded mesoporous bioactive glass nanospheres for pH-stimulated chemotherapeutic drug delivery and antibacterial susceptibility. Colloids Surf B Biointerfaces.

[49] Hollinger J, Wong MEK (1996). The integrated processes of hard tissue regeneration with special emphasis on fracture healing. Oral Surg Oral Med Oral Pathol Oral Radiol Endodontology.

[50] Luo J, Xie Z, Lam JWY, Cheng L, Chen H, Qiu C (2001). Aggregation-induced emission of 1-methyl-1,2,3,4,5-pentaphenylsilole. Chem Commun.

[51] Li D (2017). Mesoporous Bioactive Glass Functionalized with AIEgens for pH Sensing and Drug Delivery. J Bionic Eng.

[52] Wang D, Zhang C, Ren L, Li D, Yu J (2018). Biodegradable AIEgen-functionalised mesoporous bioactive glass nanoparticles for drug delivery and cell imaging. Inorg Chem Front.

[53] Xia W, Chang J, Lin J, Zhu J (2008). The pH-controlled dual-drug release from mesoporous bioactive glass/polypeptide graft copolymer nanomicelle composites. Eur J Pharm Biopharm.

[54] Chen X, Tu Y, Cheng S, Guo X, Lu T, Guo Y (2022). A colorimetric and ratiometric fluorescent paper chip for biogenic amine monitoring based on a simple pH-sensitive AIEgen. Chem Eng J.

[55] Wang X, Zhang Y, Lin C, Zhong W (2017). Sol-gel derived terbium-containing mesoporous bioactive glasses nanospheres: *In vitro* hydroxyapatite formation and drug delivery. Colloids Surf B Biointerfaces.

[56] Li R, Jia Z, Trush MA (2016). Defining ROS in Biology and Medicine. React Oxyg Species Apex NC.

[57] Sies H (2018). On the history of oxidative stress: Concept and some aspects of current development. Curr Opin Toxicol.

[58] Jones DP (2006). Redefining Oxidative Stress. Antioxid Redox Signal.

[59] Wang S, Li Y, Ma C, Huang D, Chen S, Zhu S (2023). Enzymatic molecular modification of water-soluble polyphenols: Synthesis, structure, bioactivity and application. Crit Rev Food Sci Nutr.

[60] Bendary E, Francis RR, Ali HMG, Sarwat MI, El Hady S (2013). Antioxidant and structure-activity relationships (SARs) of some phenolic and anilines compounds. Ann Agric Sci.

[61] Chen Z, Farag MA, Zhong Z, Zhang C, Yang Y, Wang S (2021). Multifaceted role of phyto-derived polyphenols in nanodrug delivery systems. Adv Drug Deliv Rev.

[62] Cazzola M, Corazzari I, Prenesti E, Bertone E, Vernè E, Ferraris S (2016). Bioactive glass coupling with natural polyphenols: Surface modification, bioactivity and anti-oxidant ability. Appl Surf Sci.

[63] Zhang X, Ferraris S, Prenesti E, Verné E (2013). Surface functionalization of bioactive glasses with natural molecules of biological significance, part II: Grafting of polyphenols extracted from grape skin. Appl Surf Sci.

[64] Gülçin İ, Huyut Z, Elmastaş M, Aboul-Enein HY (2010). Radical scavenging and antioxidant activity of tannic acid. Arab J Chem.

[65] Wang Y, luo M, Li T, Xie C, Li S, Lei B (2023). Multi-layer-structured bioactive glass nanopowder for multistage-stimulated hemostasis and wound repair. Bioact Mater.

[66] Chang H, Tian P, Hao L, Hu C, Liu B, Meng F (2024). Antioxidative bioactive glass reinforced injectable hydrogel with reactive oxygen species scavenging capacity for diabetic wounds treatment. Chem Eng J.

[67] Matter MT, Furer LA, Starsich FHL, Fortunato G, Pratsinis SE, Herrmann IK (2019). Engineering the Bioactivity of Flame-Made Ceria and Ceria/Bioglass Hybrid Nanoparticles. ACS Appl Mater Interfaces.

[68] Nicolini V, Varini E, Malavasi G, Menabue L, Menziani MC, Lusvardi G (2016). The effect of composition on structural, thermal, redox and bioactive properties of Ce-containing glasses. Mater Des.

[69] Xu W, Qin Z, Xu R, Li S, Zheng K, Tan H (2023). Injectable, pro-osteogenic and antioxidant composite microspheres composed of cerium-containing mesoporous bioactive glass and chitosan for bone regeneration applications. Ceram Int.

[70] de Castro IA, Datta RS, Ou JZ, Castellanos-Gomez A, Sriram S, Daeneke T (2017). Molybdenum Oxides - From Fundamentals to Functionality. Adv Mater.

[71] Niu W, Chen M, Guo Y, Wang M, Luo M, Cheng W (2021). A Multifunctional Bioactive Glass-Ceramic Nanodrug for Post-Surgical Infection/Cancer Therapy-Tissue Regeneration. ACS Nano.

[72] Lusvardi G, Fraulini F, D'Addato S, Zambon A (2022). Loading with Biomolecules Modulates the Antioxidant Activity of Cerium-Doped Bioactive Glasses. ACS Biomater Sci Eng.

[73] Sies H, Jones DP (2020). Reactive oxygen species (ROS) as pleiotropic physiological signalling agents. Nat Rev Mol Cell Biol.

[74] Gagge AP, Stolwijk JAJ, Hardy JD (1967). Comfort and thermal sensations and associated physiological responses at various ambient temperatures. Environ Res.

[75] Pontremoli C, Boffito M, Laurano R, Iviglia G, Torre E, Cassinelli C (2022). Mesoporous Bioactive Glasses Incorporated into an Injectable Thermosensitive Hydrogel for Sustained Co-Release of Sr2+ Ions and N-Acetylcysteine. Pharmaceutics.

[76] Chen X, Zhao Y, Geng S, Miron RJ, Zhang Q, Wu C (2015). In vivo experimental study on bone regeneration in critical bone defects using PIB nanogels/boron-containing mesoporous bioactive glass composite scaffold. Int J Nanomedicine.

[77] Jiang C, Sun G, Zhou Z, Bao Z, Lang X, Pang J (2019). Optimization of the preparation conditions of thermo-sensitive chitosan hydrogel in heterogeneous reaction using response surface methodology. Int J Biol Macromol.

[78] Couto DS, Hong Z, Mano JF (2009). Development of bioactive and biodegradable chitosan-based injectable systems containing bioactive glass nanoparticles. Acta Biomater.

[79] Wu J, Zheng K, Huang X, Liu J, Liu H, Boccaccini AldoR (2019). Thermally triggered injectable chitosan/silk fibroin/bioactive glass nanoparticle hydrogels for in-situ bone formation in rat calvarial bone defects. Acta Biomater.

[80] Moreira CDF, Carvalho SM, Sousa RG, Mansur HS, Pereira MM (2018). Nanostructured chitosan/gelatin/bioactive glass *in situ* forming hydrogel composites as a potential injectable matrix for bone tissue engineering. Mater Chem Phys.

[81] Heintzman DR, Fisher EL, Rathmell JC (2022). Microenvironmental influences on T cell immunity in cancer and inflammation. Cell Mol Immunol.

[82] Repasky EA, Evans SS, Dewhirst MW (2013). Temperature Matters!. And Why It Should Matter to Tumor Immunologists. Cancer Immunol Res.

[83] Arsalan A, Younus H (2018). Enzymes and nanoparticles: Modulation of enzymatic activity via nanoparticles. Int J Biol Macromol.

[84] Polo L, Gómez-Cerezo N, García-Fernández A, Aznar E, Vivancos J-L, Arcos D (2018). Mesoporous Bioactive Glasses Equipped with Stimuli-Responsive Molecular Gates for Controlled Delivery of Levofloxacin against Bacteria. Chem - Eur J.

[85] Polo L, Gómez-Cerezo N, Aznar E, Vivancos J-L, Sancenón F, Arcos D (2017). Molecular gates in mesoporous bioactive glasses for the treatment of bone tumors and infection. Acta Biomater.

[86] Yu X, Gou X, Wu P, Han L, Tian D, Du F (2018). Activatable Protein Nanoparticles for Targeted Delivery of Therapeutic Peptides. Adv Mater.

[87] Yang Y, Yue C, Han Y, Zhang W, He A, Zhang C (2016). Tumor-Responsive Small Molecule Self-Assembled Nanosystem for Simultaneous Fluorescence Imaging and Chemotherapy of Lung Cancer. Adv Funct Mater.

[88] Stubbs M, Aiston S, Agius L (2000). Subcellular localization, mobility, and kinetic activity of glucokinase in glucose-responsive insulin-secreting cells. Diabetes.

[89] Sui B, Zhao J, Ding T, Ruan M, Sun J, Liu X (2023). Cell membrane camouflaged mesoporous bioactive glass nanoparticles embedding glucose oxidase for enhancing targeted anti-tumor catalytic therapy. Appl Mater Today.

[90] Li F, Lu J, Kong X, Hyeon T, Ling D (2017). Dynamic Nanoparticle Assemblies for Biomedical Applications. Adv Mater.

[91] Alejo T, Uson L, Arruebo M (2019). Reversible stimuli-responsive nanomaterials with on-off switching ability for biomedical applications. J Controlled Release.

[92] Gelmi A, Schutt CE (2021). Stimuli-Responsive Biomaterials: Scaffolds for Stem Cell Control. Adv Healthc Mater.

[93] Zhao S, Mehta AS, Zhao M (2020). Biomedical applications of electrical stimulation. Cell Mol Life Sci.

[94] Ferrigno B, Bordett R, Duraisamy N, Moskow J, Arul MR, Rudraiah S (2020). Bioactive polymeric materials and electrical stimulation strategies for musculoskeletal tissue repair and regeneration. Bioact Mater.

[95] Mata D, Oliveira FJ, Neto MA, Belmonte M, Bastos AC, Lopes MA (2015). Smart electroconductive bioactive ceramics to promote in situ electrostimulation of bone. J Mater Chem B.

[96] Obata A, Nakamura S, Yamashita K (2004). Interpretation of electrical polarization and depolarization mechanisms of bioactive glasses in relation to ionic migration. Biomaterials.

[97] Mariappan CR, Roling B (2010). Mechanism and kinetics of Na ion depletion under the anode during electro-thermal poling of a bioactive glass+. J Non-Cryst Solids.

[98] Mariappan CR, Yunos DM, Boccaccini AR, Roling B (2009). Bioactivity of electro-thermally poled bioactive silicate glass. Acta Biomater.

[99] Meng D, Rath SN, Mordan N, Salih V, Kneser U, Boccaccini AR (2011). In vitro evaluation of 45S5 Bioglass®-derived glass-ceramic scaffolds coated with carbon nanotubes. J Biomed Mater Res A.

[100] Misra SK, Ohashi F, Valappil SP, Knowles JC, Roy I, Silva SRP (2010). Characterization of carbon nanotube (MWCNT) containing P(3HB)/bioactive glass composites for tissue engineering applications. Acta Biomater.

[101] Rajabi AH, Jaffe M, Arinzeh TL (2015). Piezoelectric materials for tissue regeneration: A review. Acta Biomater.

[102] Li C, Zhang S, Yao Y, Wang Y, Xiao C, Yang B (2023). Piezoelectric Bioactive Glasses Composite Promotes Angiogenesis by the Synergistic Effect of Wireless Electrical Stimulation and Active Ions. Adv Healthc Mater.

[103] Zhao F, Zhang C, Liu J, Liu L, Cao X, Chen X (2020). Periosteum structure/function-mimicking bioactive scaffolds with piezoelectric/chem/nano signals for critical-sized bone regeneration. Chem Eng J.

[104] Polley C, Distler T, Scheufler C, Detsch R, Lund H, Springer A (2023). 3D printing of piezoelectric and bioactive barium titanate-bioactive glass scaffolds for bone tissue engineering. Mater Today Bio.

[105] Thévenot J, Oliveira H, Sandre O, Lecommandoux S (2013). Magnetic responsive polymer composite materials. Chem Soc Rev.

[106] Liu G, Gao J, Ai H, Chen X (2013). Applications and Potential Toxicity of Magnetic Iron Oxide Nanoparticles. Small.

[107] Tong S, Zhu H, Bao G (2019). Magnetic iron oxide nanoparticles for disease detection and therapy. Mater Today.

[108] Wang H, Zhao S, Zhou J, Zhu K, Cui X, Huang W (2015). Biocompatibility and osteogenic capacity of borosilicate bioactive glass scaffolds loaded with Fe3O4 magnetic nanoparticles. J Mater Chem B.

[109] Li G, Feng S, Zhou D (2011). Magnetic bioactive glass ceramic in the system CaO-P2O5-SiO2-MgO-CaF2-MnO2-Fe2O3 for hyperthermia treatment of bone tumor. J Mater Sci Mater Med.

[110] Zhu Y, Shang F, Li B, Dong Y, Liu Y, Lohe MR (2013). Magnetic mesoporous bioactive glass scaffolds: preparation, physicochemistry and biological properties. J Mater Chem B.

[111] Vergnaud F, Kesse X, Jacobs A, Perton F, Begin-Colin S, Mertz D (2022). Magnetic bioactive glass nano-heterostructures: a deeper insight into magnetic hyperthermia properties in the scope of bone cancer treatment. Biomater Sci.

[112] Lei Q, Chen Y, Gao S, Li J, Xiao L, Huang H (2023). Enhanced magnetothermal effect of high porous bioglass for both bone repair and antitumor therapy. Mater Des.

[113] Yazdanpanah A, Moztarzadeh F (2019). Synthesis and characterization of Barium-Iron containing magnetic bioactive glasses: The effect of magnetic component on structure and in vitro bioactivity. Colloids Surf B Biointerfaces.

[114] Da Li G, Zhou DL, Lin Y, Pan TH, Chen GS, Yin QD (2010). Synthesis and characterization of magnetic bioactive glass-ceramics containing Mg ferrite for hyperthermia. Mater Sci Eng C.

[115] Borges R, Mendonça-Ferreira L, Rettori C, Pereira ISO, Baino F, Marchi J (2021). New sol-gel-derived magnetic bioactive glass-ceramics containing superparamagnetic hematite nanocrystals for hyperthermia application. Mater Sci Eng C.

[116] Li G, Zhang K, Pei Z, Zhang N, Yu Y, Zhao S (2019). A novel method to enhance magnetic property of bioactive glass-ceramics for hyperthermia. Ceram Int.

[117] Leenakul W, Ruangsuriya J, Jantaratana P, Pengpat K (2013). Fabrication and characterization of ferrimagnetic bioactive glass-ceramic containing BaFe12O19. Ceram Int.

[118] Adedoyin AA, Ekenseair AK (2018). Biomedical applications of magneto-responsive scaffolds. Nano Res.

[119] Liu X, Zhang Y, Wang Y, Zhu W, Li G, Ma X (2020). Comprehensive understanding of magnetic hyperthermia for improving antitumor therapeutic efficacy. Theranostics.

[120] Ying W, Zhang Y, Gao W, Cai X, Wang G, Wu X (2020). Hollow Magnetic Nanocatalysts Drive Starvation-Chemodynamic-Hyperthermia Synergistic Therapy for Tumor. ACS Nano.

[121] Chang M, Hou Z, Wang M, Li C, Lin J (2021). Recent Advances in Hyperthermia Therapy-Based Synergistic Immunotherapy. Adv Mater.

[122] Kim KS, Kim J, Lee JY, Matsuda S, Hideshima S, Mori Y (2016). Stimuli-responsive magnetic nanoparticles for tumor-targeted bimodal imaging and photodynamic/hyperthermia combination therapy. Nanoscale.

[123] Kim S, Roe DG, Choi YY, Woo H, Park J, Lee JI (2021). Artificial stimulus-response system capable of conscious response. Sci Adv.

[124] Zheng Q, Xu C, Jiang Z, Zhu M, Chen C, Fu F (2021). Smart Actuators Based on External Stimulus Response. Front Chem.

[125] Fernández M, Orozco J (2021). Advances in Functionalized Photosensitive Polymeric Nanocarriers. Polymers.

[126] Kopyshev A, Galvin CJ, Patil RR, Genzer J, Lomadze N, Feldmann D (2016). Light-Induced Reversible Change of Roughness and Thickness of Photosensitive Polymer Brushes. ACS Appl Mater Interfaces.

[127] Wang L, Long NJ, Li L, Lu Y, Li M, Cao J (2018). Multi-functional bismuth-doped bioglasses: combining bioactivity and photothermal response for bone tumor treatment and tissue repair. Light Sci Appl.

[128] de Melo C, Jullien M, Battie Y, En Naciri A, Ghanbaja J, Montaigne F (2018). Tunable Localized Surface Plasmon Resonance and Broadband Visible Photoresponse of Cu Nanoparticles/ZnO Surfaces. ACS Appl Mater Interfaces.

[129] Watanabe K, Menzel D, Nilius N, Freund H-J (2006). Photochemistry on Metal Nanoparticles. Chem Rev.

[130] Liu Y, Li T, Ma H, Zhai D, Deng C, Wang J (2018). 3D-printed scaffolds with bioactive elements-induced photothermal effect for bone tumor therapy. Acta Biomater.

[131] Du J, Ding H, Fu S, Li D, Yu B (2023). Bismuth-coated 80S15C bioactive glass scaffolds for photothermal antitumor therapy and bone regeneration. Front Bioeng Biotechnol.

[132] Kargozar S, Mozafari M, Ghodrat S, Fiume E, Baino F (2021). Copper-containing bioactive glasses and glass-ceramics: From tissue regeneration to cancer therapeutic strategies. Mater Sci Eng C.

[133] Wang H, Zeng X, Pang L, Wang H, Lin B, Deng Z (2020). Integrative treatment of anti-tumor/bone repair by combination of MoS2 nanosheets with 3D printed bioactive borosilicate glass scaffolds. Chem Eng J.

[134] Jayalekshmi AC, Sharma CP (2015). Gold nanoparticle incorporated polymer/bioactive glass composite for controlled drug delivery application. Colloids Surf B Biointerfaces.

[135] Yin J, Pan S, Guo X, Gao Y, Zhu D, Yang Q (2021). Nb2C MXene-Functionalized Scaffolds Enables Osteosarcoma Phototherapy and Angiogenesis/Osteogenesis of Bone Defects. Nano-Micro Lett.

[136] Liu S, Han Z, Hao J-N, Zhang D, Li X, Cao Y (2023). Engineering of a NIR-activable hydrogel-coated mesoporous bioactive glass scaffold with dual-mode parathyroid hormone derivative release property for angiogenesis and bone regeneration. Bioact Mater.

[137] Dang W, Li T, Li B (2018). A bifunctional scaffold with CuFeSe2 nanocrystals for tumor therapy and bone reconstruction. Biomaterials.

[138] Sun B, Findikoglu AT, Sykora M, Werder DJ, Klimov VI (2009). Hybrid Photovoltaics Based on Semiconductor Nanocrystals and Amorphous Silicon. Nano Lett.

[139] Lin H-M, Wang W-K, Hsiung P-A, Shyu S-G (2010). Light-sensitive intelligent drug delivery systems of coumarin-modified mesoporous bioactive glass. Acta Biomater.

[140] Xiang H, Yang Q, Gao Y, Zhu D, Pan S, Xu T (2020). Cocrystal Strategy toward Multifunctional 3D-Printing Scaffolds Enables NIR-Activated Photonic Osteosarcoma Hyperthermia and Enhanced Bone Defect Regeneration. Adv Funct Mater.

[141] Dang W, Jin Y, Yi K, Ju E, Zhuo C, Wei H (2021). Hemin particles-functionalized 3D printed scaffolds for combined photothermal and chemotherapy of osteosarcoma. Chem Eng J.

[142] Su X, Lyu Z, Wu Y, Gu Y-H, Huo S, Zhou C (2023). Strontium-doped bioactive glass/PDA functionalized polyetheretherketone with immunomodulatory property for enhancing photothermal clearance of Staphylococcus aureus. Mater Des.

[143] Xue Y, Niu W, Wang M, Chen M, Guo Y, Lei B (2020). Engineering a Biodegradable Multifunctional Antibacterial Bioactive Nanosystem for Enhancing Tumor Photothermo-Chemotherapy and Bone Regeneration. ACS Nano.

[144] Zhou L, Xi Y, Xue Y, Wang M, Liu Y, Guo Y (2019). Injectable Self-Healing Antibacterial Bioactive Polypeptide-Based Hybrid Nanosystems for Efficiently Treating Multidrug Resistant Infection, Skin-Tumor Therapy, and Enhancing Wound Healing. Adv Funct Mater.

[145] Kumar R, Nyk M, Ohulchanskyy TY, Flask CA, Prasad PN (2009). Combined Optical and MR Bioimaging Using Rare Earth Ion Doped NaYF4 Nanocrystals. Adv Funct Mater.

[146] Escudero A, Becerro AI, Carrillo-Carrión C, Núñez NO, Zyuzin MV, Laguna M (2017). Rare earth based nanostructured materials: synthesis, functionalization, properties and bioimaging and biosensing applications. Nanophotonics.

[147] Hemmer E, Venkatachalam N, Hyodo H, Hattori A, Ebina Y, Kishimoto H (2013). Upconverting and NIR emitting rare earth based nanostructures for NIR-bioimaging. Nanoscale.

[148] Ma L, Zhou Y, Zhang Z, Liu Y, Zhai D, Zhuang H (2020). Multifunctional bioactive Nd-Ca-Si glasses for fluorescence thermometry, photothermal therapy, and burn tissue repair. Sci Adv.

[149] Huang WM, Song CL, Fu YQ, Wang CC, Zhao Y, Purnawali H (2013). Shaping tissue with shape memory materials. Adv Drug Deliv Rev.

[150] Sun L, Huang WM, Ding Z, Zhao Y, Wang CC, Purnawali H (2012). Stimulus-responsive shape memory materials: A review. Mater Des.

[151] Liverani L, Liguori A, Zezza P, Gualandi C, Toselli M, Boccaccini AR (2022). Nanocomposite electrospun fibers of poly(ε-caprolactone)/bioactive glass with shape memory properties. Bioact Mater.

[152] Leite ÁJ, Caridade SG, Mano JF (2016). Synthesis and characterization of bioactive biodegradable chitosan composite spheres with shape memory capability. J Non-Cryst Solids.

[153] Correia CO, Leite ÁJ, Mano JF (2015). Chitosan/bioactive glass nanoparticles scaffolds with shape memory properties. Carbohydr Polym.

[154] Wang L, Qiu Y, Guo Y, Si Y, Liu L, Cao J (2019). Smart, Elastic, and Nanofiber-Based 3D Scaffolds with Self-Deploying Capability for Osteoporotic Bone Regeneration. Nano Lett.

[155] Yao L, Gao H, Lin Z, Dai Q, Zhu S, Li S (2022). A shape memory and antibacterial cryogel with rapid hemostasis for noncompressible hemorrhage and wound healing. Chem Eng J.

[156] Zhao F, Yang Z, Xiong H, Yan Y, Chen X, Shao L (2023). A bioactive glass functional hydrogel enhances bone augmentation via synergistic angiogenesis, self-swelling and osteogenesis. Bioact Mater.

[157] Miao S, Castro N, Nowicki M, Xia L, Cui H, Zhou X (2017). 4D printing of polymeric materials for tissue and organ regeneration. Mater Today.

[158] Verma AS, Singh A, Kumar D, Dubey AK (2020). Electro-mechanical and Polarization-Induced Antibacterial Response of 45S5 Bioglass-Sodium Potassium Niobate Piezoelectric Ceramic Composites. ACS Biomater Sci Eng.

[159] Verma AS, Kumar D, Dubey AK (2020). Antibacterial and cellular response of piezoelectric Na0.5K0.5NbO3modified 1393 bioactive glass. Mater Sci Eng C.

[160] Zhang X, Wei H, Dong C, Wang J, Zhang T, Huang L (2023). 3D printed hydrogel/bioceramics core/shell scaffold with NIR-II triggered drug release for chemo-photothermal therapy of bone tumors and enhanced bone repair. Chem Eng J.

[161] Miguez-Pacheco V, Hench LL, Boccaccini AR (2015). Bioactive glasses beyond bone and teeth: Emerging applications in contact with soft tissues. Acta Biomater.

[162] Jia W, Lau GY, Huang W, Zhang C, Tomsia AP, Fu Q (2015). Bioactive Glass for Large Bone Repair. Adv Healthc Mater.

[163] Liao J, Han R, Wu Y, Qian Z (2021). Review of a new bone tumor therapy strategy based on bifunctional biomaterials. Bone Res.

[164] Feng L, Dong Z, Tao D, Zhang Y, Liu Z (2018). The acidic tumor microenvironment: a target for smart cancer nano-theranostics. Natl Sci Rev.

[165] Zhang Y, Wang X, Su Y, Chen D, Zhong W (2016). A doxorubicin delivery system: Samarium/mesoporous bioactive glass/alginate composite microspheres. Mater Sci Eng C.

[166] Mehnath S, Karthikeyan K, Rajan M, Jeyaraj M (2021). Fabrication of bone-targeting hyaluronic acid coupled alendronate-bioactive glass for osteosarcoma therapy. Mater Chem Phys.

[167] Zhang Y, Zhang W, Zhang X, Zhou Y (2023). Erbium-ytterbium containing upconversion mesoporous bioactive glass microspheres for tissue engineering: luminescence monitoring of biomineralization and drug release. Acta Biomater.

[168] Lei P, Feng J, Zhang H (2020). Emerging biomaterials: Taking full advantage of the intrinsic properties of rare earth elements. Nano Today.

[169] Tan G-R, Wang M, Hsu C-Y, Chen N, Zhang Y (2016). Small Upconverting Fluorescent Nanoparticles for Biosensing and Bioimaging. Adv Opt Mater.

[170] Zhang W, Zhang X, Zhou Y, Zhang Y (2023). Luminescence biomonitoring and antibacterial properties of Er3+-doped SiO2-CaO-P2O5 bioactive glass microspheres. Ceram Int.

[171] Xue Y, Du Y, Yan J, Liu Z, Ma PX, Chen X (2015). Monodisperse photoluminescent and highly biocompatible bioactive glass nanoparticles for controlled drug delivery and cell imaging. J Mater Chem B.

[172] Wang F, Zhai D, Wu C, Chang J (2016). Multifunctional mesoporous bioactive glass/upconversion nanoparticle nanocomposites with strong red emission to monitor drug delivery and stimulate osteogenic differentiation of stem cells. Nano Res.

[173] Chen M, Wang M, Niu W, Cheng W, Guo Y, Wang Y (2021). Multifunctional Protein-Decorated Bioactive Glass Nanoparticles for Tumor-Specific Therapy and Bioimaging In Vitro and In Vivo. ACS Appl Mater Interfaces.

[174] Qin X, Wu C, Niu D, Qin L, Wang X, Wang Q (2021). Peroxisome inspired hybrid enzyme nanogels for chemodynamic and photodynamic therapy. Nat Commun.

[175] Huang L, Zhao S, Wu J, Yu L, Singh N, Yang K (2021). Photodynamic therapy for hypoxic tumors: Advances and perspectives. Coord Chem Rev.

[176] Pang L, Zhao R, Chen J, Ding J, Chen X, Chai W (2022). Osteogenic and anti-tumor Cu and Mn-doped borosilicate nanoparticles for syncretic bone repair and chemodynamic therapy in bone tumor treatment. Bioact Mater.

[177] Huang R, Ni N, Gu L, Su Y, Ju Y, Zhang D (2024). Chargeable persistent luminescence 3D-printed scaffolds: A stepwise tactic for osteosarcoma treatment. Chem Eng J.

[178] Huang Z, Tian Z, Zhu M, Wu C, Zhu Y (2021). Recent Advances in Biomaterial Scaffolds for Integrative Tumor Therapy and Bone Regeneration. Adv Ther.

[179] Gao X, Li L, Cai X, Huang Q, Xiao J, Cheng Y (2021). Targeting nanoparticles for diagnosis and therapy of bone tumors: Opportunities and challenges. Biomaterials.

[180] Yang Z, Zhao F, Zhang W, Yang Z, Luo M, Liu L (2021). Degradable photothermal bioactive glass composite hydrogel for the sequential treatment of tumor-related bone defects: From anti-tumor to repairing bone defects. Chem Eng J.

[181] Liu Y, Lin R, Ma L, Zhuang H, Feng C, Chang J (2020). Mesoporous bioactive glass for synergistic therapy of tumor and regeneration of bone tissue. Appl Mater Today.

[182] Liang B, Zuo D, Yu K, Cai X, Qiao B, Deng R (2020). Multifunctional bone cement for synergistic magnetic hyperthermia ablation and chemotherapy of osteosarcoma. Mater Sci Eng C.

[183] Danewalia SS, Singh K (2021). Bioactive glasses and glass-ceramics for hyperthermia treatment of cancer: state-of-art, challenges, and future perspectives. Mater Today Bio.

[184] Wei H, Cui J, Lin K, Xie J, Wang X (2022). Recent advances in smart stimuli-responsive biomaterials for bone therapeutics and regeneration. Bone Res.

[185] Han X, Yi W, Chen S, Cai Z, Zhu Y, Han W (2023). Ultrasound-responsive smart composite biomaterials in tissue repair. Nano Today.

[186] Levingstone T, Ali B, Kearney C, Dunne N (2021). Hydroxyapatite sonosensitization of ultrasound-triggered, thermally responsive hydrogels: An on-demand delivery system for bone repair applications. J Biomed Mater Res B Appl Biomater.

[187] Osminkina LA, Nikolaev AL, Sviridov AP, Andronova NV, Tamarov KP, Gongalsky MB (2015). Porous silicon nanoparticles as efficient sensitizers for sonodynamic therapy of cancer. Microporous Mesoporous Mater.

[188] Villanueva J, Herlyn M (2008). Melanoma and the tumor microenvironment. Curr Oncol Rep.

[189] Umansky V, Sevko A (2012). Overcoming immunosuppression in the melanoma microenvironment induced by chronic inflammation. Cancer Immunol Immunother.

[190] Marzagalli M, Ebelt ND, Manuel ER (2019). Unraveling the crosstalk between melanoma and immune cells in the tumor microenvironment. Semin Cancer Biol.

[191] Yuan C, Zhang D, Tang Y, Guo Z, Lin K, Yu Y (2023). Fibrous dressing containing bioactive glass with combined chemotherapy and wound healing promotion for post-surgical treatment of melanoma. Biomater Adv.

[192] Zhao X, Lang Q, Yildirimer L, Lin ZY, Cui W, Annabi N (2016). Photocrosslinkable Gelatin Hydrogel for Epidermal Tissue Engineering. Adv Healthc Mater.

[193] Liu X, Shen M, Bing T, Zhang X, Li Y, Cai Q (2024). A Bioactive Injectable Hydrogel Regulates Tumor Metastasis and Wound Healing for Melanoma via NIR-Light Triggered Hyperthermia. Adv. Sci.

[194] Huang H, Wang X, Wang W, Qu X, Song X, Zhang Y (2022). Injectable hydrogel for postoperative synergistic photothermal-chemodynamic tumor and anti-infection therapy. Biomaterials.

[195] Chen M, Winston DD, Wang M, Niu W, Cheng W, Guo Y (2022). Hierarchically multifunctional bioactive nanoglass for integrated tumor/infection therapy and impaired wound repair. Mater Today.

[196] del Rosal B, Carrasco E, Ren F, Benayas A, Vetrone F, Sanz-Rodríguez F (2016). Infrared-Emitting QDs for Thermal Therapy with Real-Time Subcutaneous Temperature Feedback. Adv Funct Mater.

[197] Ashrafizadeh M, Zarrabi A, Bigham A, Taheriazam A, Saghari Y, Mirzaei S (2023). (Nano)platforms in breast cancer therapy: Drug/gene delivery, advanced nanocarriers and immunotherapy. Med Res Rev.

[198] Zhu L, Zhong Y, Wu S, Yan M, Cao Y, Mou N (2022). Cell membrane camouflaged biomimetic nanoparticles: Focusing on tumor theranostics. Mater Today Bio.

[199] Xu C-H, Ye P-J, Zhou Y-C, He D-X, Wei H, Yu C-Y (2020). Cell membrane-camouflaged nanoparticles as drug carriers for cancer therapy. Acta Biomater.

[200] Dhas N, García MC, Kudarha R, Pandey A, Nikam AN, Gopalan D (2022). Advancements in cell membrane camouflaged nanoparticles: A bioinspired platform for cancer therapy. J Controlled Release.

[201] Zhang J, Sun Y, Ren L, Chen L, Nie L, Shavandi A (2023). Red Blood Cell Membrane-Camouflaged Polydopamine and Bioactive Glass Composite Nanoformulation for Combined Chemo/Chemodynamic/Photothermal Therapy. ACS Biomater Sci Eng.

[202] Liang Y, Zhang H, Song X, Yang Q (2020). Metastatic heterogeneity of breast cancer: Molecular mechanism and potential therapeutic targets. Semin Cancer Biol.

[203] Wang S, Wu W, Lin X, Zhang KM, Wu Q, Luo M (2023). Predictive and prognostic biomarkers of bone metastasis in breast cancer: current status and future directions. Cell Biosci.

[204] He C, Yu L, Yao H, Chen Y, Hao Y (2021). Combinatorial Photothermal 3D-Printing Scaffold and Checkpoint Blockade Inhibits Growth/Metastasis of Breast Cancer to Bone and Accelerates Osteogenesis. Adv Funct Mater.

[205] Lin B, Chen H, Liang D, Lin W, Qi X, Liu H (2019). Acidic pH and High-H2O2 Dual Tumor Microenvironment-Responsive Nanocatalytic Graphene Oxide for Cancer Selective Therapy and Recognition. ACS Appl Mater Interfaces.

[206] Wang X, Zhong X, Liu Z, Cheng L (2020). Recent progress of chemodynamic therapy-induced combination cancer therapy. Nano Today.

[207] Han Y-H, Liu C-G, Chen B-Q, Fu C-P, Kankala RK, Wang S-B (2022). Orchestrated tumor apoptosis (Cu2+) and bone tissue calcification (Ca2+) by hierarchical Copper/Calcium-ensembled bioactive silica for osteosarcoma therapy. Chem Eng J.

[208] El-Fiqi A, Kim H-W (2021). Iron ions-releasing mesoporous bioactive glass ultrasmall nanoparticles designed as ferroptosis-based bone cancer nanotherapeutics: Ultrasonic-coupled sol-gel synthesis, properties and iron ions release. Mater Lett.

[209] Lu S, Li Y, Yu Y (2024). Glutathione-Scavenging Celastrol-Cu Nanoparticles Induce Self-Amplified Cuproptosis for Augmented Cancer Immunotherapy. Adv Mater.

[210] Patel A, Sant S (2016). Hypoxic tumor microenvironment: Opportunities to develop targeted therapies. Biotechnol Adv.

[211] Petrova V, Annicchiarico-Petruzzelli M, Melino G, Amelio I (2018). The hypoxic tumour microenvironment. Oncogenesis.

[212] Xie Z, Guo W, Guo N, Huangfu M, Liu H, Lin M (2018). Targeting tumor hypoxia with stimulus-responsive nanocarriers in overcoming drug resistance and monitoring anticancer efficacy. Acta Biomater.

[213] Perche F, Biswas S, Wang T, Zhu L, Torchilin VP (2014). Hypoxia-Targeted siRNA Delivery. Angew Chem.

[214] He H, Zhu R, Sun W, Cai K, Chen Y, Yin L (2018). Selective cancer treatment via photodynamic sensitization of hypoxia-responsive drug delivery. Nanoscale.

[215] Li Y, Wang J, Tang Y, Lu S, Lv Y, Li W (2023). Stimuli-responsive ultra-small vanadate prodrug nanoparticles with NIR photothermal properties to precisely inhibit Na/K-ATPase for enhanced cancer therapy. Nanoscale.

[216] Zimmerli W (2014). Clinical presentation and treatment of orthopaedic implant-associated infection. J Intern Med.

[217] Inzana JA, Schwarz EM, Kates SL, Awad HA (2016). Biomaterials approaches to treating implant-associated osteomyelitis. Biomaterials.

[218] Dréno B, Dagnelie MA, Khammari A, Corvec S (2020). The Skin Microbiome: A New Actor in Inflammatory Acne. Am J Clin Dermatol.

[219] Ptasiewicz M, Grywalska E, Mertowska P, Korona-Głowniak I, Poniewierska-Baran A, Niedźwiedzka-Rystwej P (2022). Armed to the Teeth-The Oral Mucosa Immunity System and Microbiota. Int J Mol Sci.

[220] Mark Welch JL, Ramírez-Puebla ST, Borisy GG (2020). Oral Microbiome Geography: Micron-Scale Habitat and Niche. Cell Host Microbe.

[221] Joseph S, Curtis MA (2021). Microbial transitions from health to disease. Periodontol 2000.

[222] Besinis A, De Peralta T, Tredwin CJ, Handy RD (2015). Review of Nanomaterials in Dentistry: Interactions with the Oral Microenvironment, Clinical Applications, Hazards, and Benefits. ACS Nano.

[223] Yu K, Zhang Q, Dai Z, Zhu M, Xiao L, Zhao Z (2023). Smart Dental Materials Intelligently Responding to Oral pH to Combat Caries: A Literature Review. Polymers.

[224] He Y, Vasilev K, Zilm P (2023). pH-Responsive Biomaterials for the Treatment of Dental Caries-A Focussed and Critical Review. Pharmaceutics.

[225] Lu J, Liu Z, Wang K, Gu M, Peng X, Zhang Y (2022). Odontogenesis by Endocytosis of Peptide Embedding Bioactive Glass Composite. J Dent Res.

[226] Irshad N, Jahanzeb N, Alqasim A, Bousaleh R, Almehrij M, Ghafoor S (2023). Synthesis and analyses of injectable fluoridated-bioactive glass hydrogel for dental root canal sealing. PLOS ONE.

[227] Malik Q ul A, Iftikhar S, Zahid S, Safi SZ, Khan AF, Nawshad M (2020). Smart injectable self-setting bioceramics for dental applications. Mater Sci Eng C.

[228] Woo HN, Cho YJ, Tarafder S, Lee CH (2021). The recent advances in scaffolds for integrated periodontal regeneration. Bioact Mater.

[229] Takallu S, Mirzaei E, Zakeri Bazmandeh A, Ghaderi Jafarbeigloo HR, Khorshidi H (2024). Addressing Antimicrobial Properties in Guided Tissue/Bone Regeneration Membrane: Enhancing Effectiveness in Periodontitis Treatment. ACS Infect Dis.

[230] Hu Z, Lv X, Zhang H, Zhuang S, Zheng K, Zhou T (2024). An injectable gel based on photo-cross-linkable hyaluronic acid and mesoporous bioactive glass nanoparticles for periodontitis treatment. Int J Biol Macromol.

[231] Sanz-Sánchez I, Sanz-Martín I, Ortiz-Vigón A, Molina A, Sanz M (2022). Complications in bone-grafting procedures: Classification and management. Periodontol 2000.

[232] Ghimire A, Song J (2021). Anti-Periprosthetic Infection Strategies: From Implant Surface Topographical Engineering to Smart Drug-Releasing Coatings. ACS Appl Mater Interfaces.

[233] Depypere M, Morgenstern M, Kuehl R, Senneville E, Moriarty TF, Obremskey WT (2020). Pathogenesis and management of fracture-related infection. Clin Microbiol Infect Off Publ Eur Soc Clin Microbiol Infect Dis.

[234] Zarghami V, Ghorbani M, Bagheri KP, Shokrgozar MA (2020). In vitro bactericidal and drug release properties of vancomycin-amino surface functionalized bioactive glass nanoparticles. Mater Chem Phys.

[235] Lv X, Zhang J, Yang D, Shao J, Wang W, Zhang Q (2020). Recent advances in pH-responsive nanomaterials for anti-infective therapy. J Mater Chem B.

[236] Zhang R, Ding J, Lu X, Yao A, Wang D (2023). pH-responsive drug release and antibacterial activity of chitosan-coated core/shell borate glass-hydroxyapatite microspheres. Ceram Int.

[237] Han L, Huang Z, Zhu M, Zhu Y, Li H (2022). Drug-loaded zeolite imidazole framework-8-functionalized bioglass scaffolds with antibacterial activity for bone repair. Ceram Int.

[238] Li R, Chen T, Lu J, Hu H, Zheng H, Zhu P (2023). Metal-organic frameworks doped with metal ions for efficient sterilization: Enhanced photocatalytic activity and photothermal effect. Water Res.

[239] Xu J-W, Yao K, Xu Z-K (2019). Nanomaterials with a photothermal effect for antibacterial activities: an overview. Nanoscale.

[240] Wang C, Chen Q, Yin R, Yuan X, Kang H, Cai A (2024). Photothermal effect and antimicrobial properties of cerium-doped bioactive glasses. Ceram Int.

[241] Maleki A, He J, Bochani S, Nosrati V, Shahbazi M-A, Guo B (2021). Multifunctional Photoactive Hydrogels for Wound Healing Acceleration. ACS Nano.

[242] Hou Q, Liu K, Lian C, Liu J, Wei W, Qiu T (2023). A Gelatin-Based Composite Hydrogel with a “One Stone, Two Birds” Strategy for Photothermal Antibacterial and Vascularization of Infected Wounds. Biomacromolecules.

[243] Rauf A, Ye J, Zhang S, Qi Y, Wang G, Che Y (2019). Copper(II)-based coordination polymer nanofibers as a highly effective antibacterial material with a synergistic mechanism. Dalton Trans.

[244] Li T, Wang Y, Lei B (2024). Photothermal-antibacterial bioactive noncrystalline nanosystem promotes infected wound tissue regeneration through thermo-ions activation. Chem Eng J.

[245] Zhang M, Fan Z, Zhang J, Yang Y, Huang C, Zhang W (2023). Multifunctional chitosan/alginate hydrogel incorporated with bioactive glass nanocomposites enabling photothermal and nitric oxide release activities for bacteria-infected wound healing. Int J Biol Macromol.

[246] Qu L, Yang L, Ren Y, Ren X, Fan D, Xu K (2020). A signal-off electrochemical sensing platform based on Fe3S4-Pd and pineal mesoporous bioactive glass for procalcitonin detection. Sens Actuators B Chem.

[247] Mandakhbayar N, Ji Y, El-Fiqi A, Patel KD, Yoon DS, Dashnyam K (2024). Double hits with bioactive nanozyme based on cobalt-doped nanoglass for acute and diabetic wound therapies through anti-inflammatory and pro-angiogenic functions. Bioact Mater.

[248] Zhang Y, Hu M, Zhang W, Zhang X (2022). Construction of tellurium-doped mesoporous bioactive glass nanoparticles for bone cancer therapy by promoting ROS-mediated apoptosis and antibacterial activity. J Colloid Interface Sci.

[249] Tian P, Zhao L, Kim J, Li X, Liu C, Cui X (2023). Dual stimulus responsive borosilicate glass (BSG) scaffolds promote diabetic alveolar bone defectsrepair by modulating macrophage phenotype. Bioact Mater.

[250] Zhao H, Huang J, Li Y, Lv X, Zhou H, Wang H (2020). ROS-scavenging hydrogel to promote healing of bacteria infected diabetic wounds. Biomaterials.

[251] Wang W, Zheng J, Hong X, Zhou J, Xiong Y, Yang H (2024). Micro-environment triple-responsive hyaluronic acid hydrogel dressings to promote antibacterial activity, collagen deposition, and angiogenesis for diabetic wound healing. J Mater Chem B.

[252] Li H, Li B, Lv D, Li W, Lu Y, Luo G (2023). Biomaterials releasing drug responsively to promote wound healing via regulation of pathological microenvironment. Adv Drug Deliv Rev.

[253] Xu B, Cao Q, Zhang Y, Yu W, Zhu J, Liu D (2018). Microneedles Integrated with ZnO Quantum-Dot-Capped Mesoporous Bioactive Glasses for Glucose-Mediated Insulin Delivery. ACS Biomater Sci Eng.

[254] Jiang G, Xu B, Zhu J, Zhang Y, Liu T, Song G (2019). Polymer microneedles integrated with glucose-responsive mesoporous bioactive glass nanoparticles for transdermal delivery of insulin. Biomed Phys Eng Express.

[255] Xing C, Zhu H, Dou X, Gao L, Baddi S, Zou Y (2023). Infected Diabetic Wound Regeneration Using Peptide-Modified Chiral Dressing to Target Revascularization. ACS Nano.

[256] Zhang S, Ge G, Qin Y, Li W, Dong J, Mei J (2023). Recent advances in responsive hydrogels for diabetic wound healing. Mater Today Bio.

[257] Moura J, Madureira P, Leal EC, Fonseca AC, Carvalho E (2019). Immune aging in diabetes and its implications in wound healing. Clin Immunol Orlando Fla.

[258] Louiselle AE, Niemiec SM, Zgheib C, Liechty KW (2021). Macrophage polarization and diabetic wound healing. Transl Res J Lab Clin Med.

[259] Daryabor G, Atashzar MR, Kabelitz D, Meri S, Kalantar K (2020). The Effects of Type 2 Diabetes Mellitus on Organ Metabolism and the Immune System. Front Immunol.

[260] Zeng H, Ying Z-R, Luo X, Tan S, Liu X-H, Zhao X-Y (2023). Gallic acid-modified bioglass with combined photothermal and antibacterial effects for the regeneration of infected diabetic wound. Compos Part B Eng.

[261] Zhu S, Li M, Wang Z, Feng Q, Gao H, Li Q (2023). Bioactive Glasses-Based Nanozymes Composite Macroporous Cryogel with Antioxidative, Antibacterial, and Pro-Healing Properties for Diabetic Infected Wound Repair. Adv Healthc Mater.

[262] Kocher T, König J, Borgnakke WS, Pink C, Meisel P (2018). Periodontal complications of hyperglycemia/diabetes mellitus: Epidemiologic complexity and clinical challenge. Periodontol 2000.

[263] Xu Z, Qi X, Bao M, Zhou T, Shi J, Xu Z (2023). Biomineralization inspired 3D printed bioactive glass nanocomposite scaffolds orchestrate diabetic bone regeneration by remodeling micromilieu. Bioact Mater.

[264] Wu Z, Bai J, Ge G, Wang T, Feng S, Ma Q (2022). Regulating Macrophage Polarization in High Glucose Microenvironment Using Lithium-Modified Bioglass-Hydrogel for Diabetic Bone Regeneration. Adv Healthc Mater.

[265] Knight ET, Liu J, Seymour GJ, Faggion CM, Cullinan MP (2016). Risk factors that may modify the innate and adaptive immune responses in periodontal diseases. Periodontol 2000.

[266] Chen Y, Chen L, Wang Y, Lin K, Liu J (2022). Lithium-containing bioactive glasses enhanced 3D-printed PLGA scaffolds for bone regeneration in diabetes. Compos Part B Eng.

[267] Hickman DA, Pawlowski CL, Sekhon UDS, Marks J, Gupta AS (2018). Biomaterials and Advanced Technologies for Hemostatic Management of Bleeding. Adv Mater.

[268] Dong R, Zhang H, Guo B (2022). Emerging hemostatic materials for non-compressible hemorrhage control. Natl Sci Rev.

[269] Curry N, Hopewell S, Dorée C, Hyde C, Brohi K, Stanworth S (2011). The acute management of trauma hemorrhage: a systematic review of randomized controlled trials. Crit Care.

[270] Lu X, Liu Z, Jia Q, Wang Q, Zhang Q, Li X (2023). Flexible Bioactive Glass Nanofiber-Based Self-Expanding Cryogels with Superelasticity and Bioadhesion Enabling Hemostasis and Wound Healing. ACS Nano.

[271] Zhu Y, Wang Y, Xia G, Zhang X, Deng S, Zhao X (2023). Oral Delivery of Bioactive Glass-Loaded Core-Shell Hydrogel Microspheres for Effective Treatment of Inflammatory Bowel Disease. Adv Sci.

[272] Park KC, Gaze DC, Collinson PO, Marber MS (2017). Cardiac troponins: from myocardial infarction to chronic disease. Cardiovasc Res.

[273] Ellahham S (2020). Artificial Intelligence: The Future for Diabetes Care. Am J Med.

[274] Bidart JM, Thuillier F, Augereau C, Chalas J, Daver A, Jacob N (1999). Kinetics of serum tumor marker concentrations and usefulness in clinical monitoring. Clin Chem.

[275] Janse van Rensburg HJ, Spiliopoulou P, Siu LL (2022). Circulating Biomarkers for Therapeutic Monitoring of Anti-cancer Agents. The Oncologist.

[276] Lui YS, Sow WT, Tan LP, Wu Y, Lai Y, Li H (2019). 4D printing and stimuli-responsive materials in biomedical aspects. Acta Biomater.

[277] Qu M, Wang C, Zhou X, Libanori A, Jiang X, Xu W (2021). Multi-Dimensional Printing for Bone Tissue Engineering. Adv Healthc Mater.

[278] Miri AK, Khalilpour A, Cecen B, Maharjan S, Shin SR, Khademhosseini A (2019). Multiscale bioprinting of vascularized models. Biomaterials.

[279] Li Y-C, Zhang YS, Akpek A, Shin SR, Khademhosseini A (2016). 4D bioprinting: the next-generation technology for biofabrication enabled by stimuli-responsive materials. Biofabrication.

[280] Wang Y, Cui H, Esworthy T, Mei D, Wang Y, Zhang LG (2022). Emerging 4D Printing Strategies for Next-Generation Tissue Regeneration and Medical Devices. Adv Mater.

[281] Joshi S, Rawat K, C K, Rajamohan V, Mathew AT, Koziol K (2020). 4D printing of materials for the future: Opportunities and challenges. Appl Mater Today.

